# Pyrazolo[4,3-c]pyridine Sulfonamides as Carbonic Anhydrase Inhibitors: Synthesis, Biological and In Silico Studies

**DOI:** 10.3390/ph15030316

**Published:** 2022-03-07

**Authors:** Andrea Angeli, Victor Kartsev, Anthi Petrou, Boris Lichitsky, Andrey Komogortsev, Mariana Pinteala, Athina Geronikaki, Claudiu T. Supuran

**Affiliations:** 1 Sezione di Scienze Farmaceutiche, NeuroFarba Department, Universita degli Studi di Firenze, Via Ugo Schiff 6, 50019 Sesto Fiorentino, Italy; andrea.angeli@unifi.it; 2 Centre of Advanced Research in Bionanoconjugates and Biopolymers, Petru Poni Institute of Macromolecular Chemistry, Aleea Grigore Ghica-Voda, no. 41A, 700487 Iasi, Romania; pinteala@icmpp.ro; 3 InterBioScreen, 142432 Chernogolovka, Russia; vkartsev@ibscreen.chg.ru; 4 Department of Pharmacy, School of Health, Aristotle University of Thessaloniki, 54124 Thessaloniki, Greece; anthi.petrou.thessaloniki1@gmail.com; 5 Zelinsky Institute of Organic Chemistry, Leninsky Prospect, 119991 Moscow, Russia; blich2006@mail.ru (B.L.); dna5@mail.ru (A.K.)

**Keywords:** carbonic anhydrases, CA inhibitors, 3β and 3γCAs, docking, cytotoxicity

## Abstract

Carbonic anhydrases (CAs, EC 4.2.1.1) catalyze the essential reaction of CO_2_ hydration in all living organisms, being actively involved in the regulation of a plethora of patho-/physiological conditions. A series of chromene-based sulfonamides were synthesized and tested as possible CA inhibitors. On the other hand, in microorganisms, the β- and γ- classes are expressed in addition to the α- class, showing substantial structural differences to the human isoforms. In this scenario, not only human but also bacterial CAs are of particular interest as new antibacterial agents with an alternative mechanism of action for fighting the emerging problem of extensive drug resistance afflicting most countries worldwide. Pyrazolo[4,3-c]pyridine sulfonamides were synthesized using methods of organic chemistry. Their inhibitory activity, assessed against the cytosolic human isoforms hCA I and hCA II, the transmembrane hCA IX and XII, and β- and γ-CAs from three different bacterial strains, was evaluated by a stopped-flow CO_2_ hydrase assay. Several of the investigated derivatives showed interesting inhibition activity towards the cytosolic associate isoforms hCA I and hCA II, as well as the 3β- and 3γ-CAs. Furthermore, computational procedures were used to investigate the binding mode of this class of compounds within the active site of hCA IX. Four compounds (**1f**, **1g**, **1h** and **1k**) were more potent than AAZ against hCA I. Furthermore, compound **1f** also showed better activity than AAZ against the hCA II isoform. Moreover, ten compounds out of eleven appeared to be very potent against the γ-CA from *E.coli,* with a Ki much lower than that of the reference drug. Most of the compounds showed better activity than AAZ against hCA I as well as the γ-CA from *E.coli* and the β-CA from *Burkholderia pseudomallei (BpsCAβ).* Compounds **1f** and **1k** showed a good selectivity index against hCA I and hCA XII, while **1b** was selective against all 3β-CA isoforms from *E.coli*, *BpsCA*, and *VhCA* and all 3γ-CA isoforms from *E.coli*, *BpsCA* and *PgiCA.*

## 1. Introduction

Carbonic anhydrases (CAs, EC 4.2.1.1) are ubiquitous metalloenzymes, present throughout most living organisms and encoded by eight evolutionarily unrelated gene families: the α-, β-, γ-, δ-, ζ-, η-, θ-, and ι-CAs [1,2,3]. All these enzymes catalyze the reversible hydration of carbon dioxide to a bicarbonate ion and proton (CO_2_ + H_2_O ⇆ HCO_3_^−^ + H^+^), which is essential in a variety of physiological processes [3,4], and it has been shown that abnormal levels or activities of these enzymes are often associated with different human diseases [3]. All human CAs (hCAs) belong to the α-class, and, to date, fifteen isoforms have been discovered, which differ by molecular features, oligomeric arrangement, cellular localization, distribution in organs and tissues, expression levels, kinetic properties, and response to different classes of inhibitors [5]. On the other hand, in microorganisms, the β- and γ- classes are expressed in addition to the α- class, showing substantial structural differences to the human isoforms. In this scenario, bacterial CAs are of particular interest due to the fact that their inhibition leads to impaired bacterial growth (bacteriostatic or bactericidal effects), reduces the expression of virulence factors, and furnishes an alternative option in combination with the current therapeutically used drugs [2].

**Figure 1:**
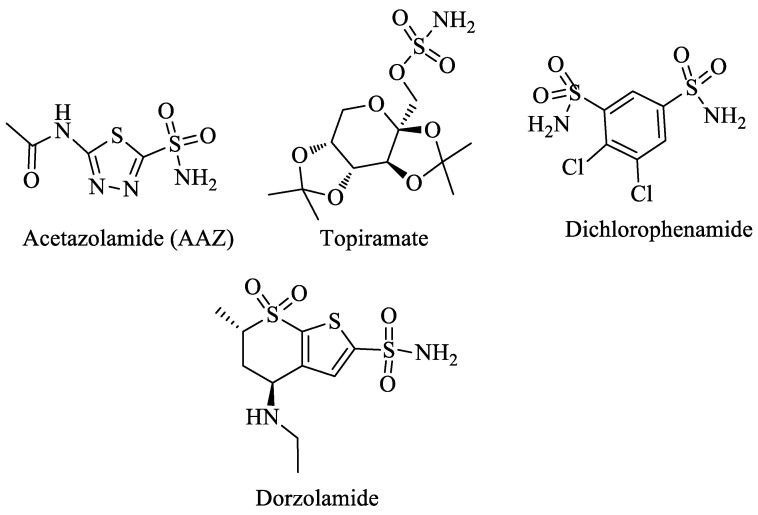
Known CA inhibitors.

Some of the CA inhibitors mentioned in ChEMBL are presented in Figure 1.

**Figure 2:**
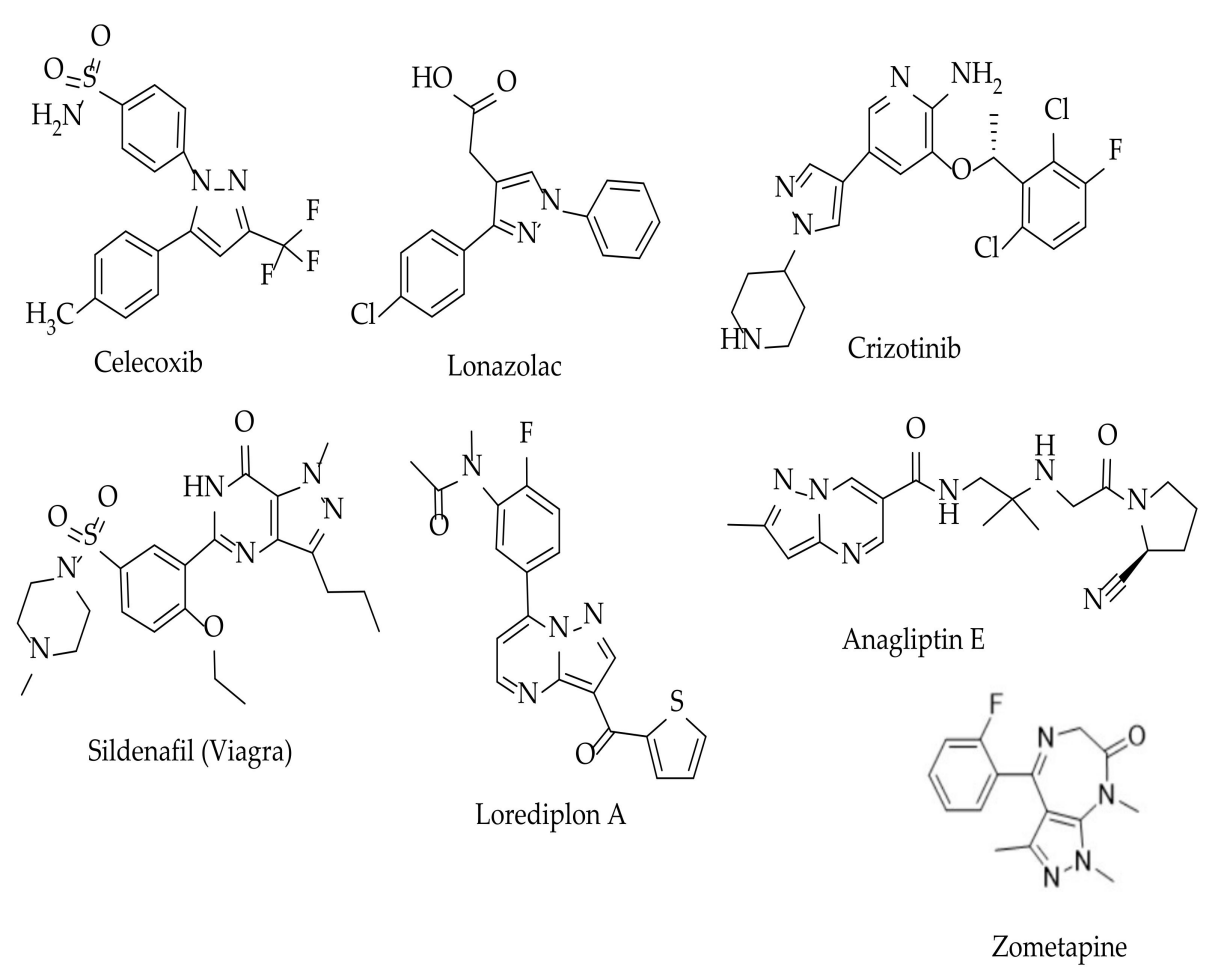
Structure of drugs bearing the pyrazole moiety.

In this context, the pyrazole scaffold is an adaptable molecule that has attracted the interest of medicinal chemists due to its wide range of various pharmacological activities, being a structural motif present in several drug molecules. These molecules are: celecoxib and lonazolac, approved COX-2 inhibitor drugs [6,7]; crizotinib [8], an anticancer drug; sildenafil [8] (Viagra), a PDE5 inhibitor; zometapine [9], an antidepressant; lorediplon A, used for the treatment of insomnia [10]; anagliptin E, an inhibitor of dipeptidyl peptidase-4 (DPP-4) for the treatment of type 2 diabetes mellitus [11] (Figure 2); and many others. Furthermore, their derivatives are reported to possess antimicrobial [12,13,14], antiviral [15,16,17], antidiabetic [18,19], anti-Alzheimer [20,21], antitubercular [22,23], and antileishmanial [24] properties, as well as α-glucosidase inhibitory activity [25].

On the other hand, pyrazolopyridine derivatives are another interesting scaffold and have appeared in many medicinal chemistry programs due to their great variety of biological activities. These derivatives possess antimicrobial [26,27], antioxidant [28], anxiolytic [29], anticancer [30,31], antiproliferative [32], cytotoxic [33], antileishmanial [34,35], and antimalarial [36] properties, as well as phosphodiesterrase (PDE4) [37], kinase [38], and angiogenesis [39] inhibitory activities.

**Figure 3:**
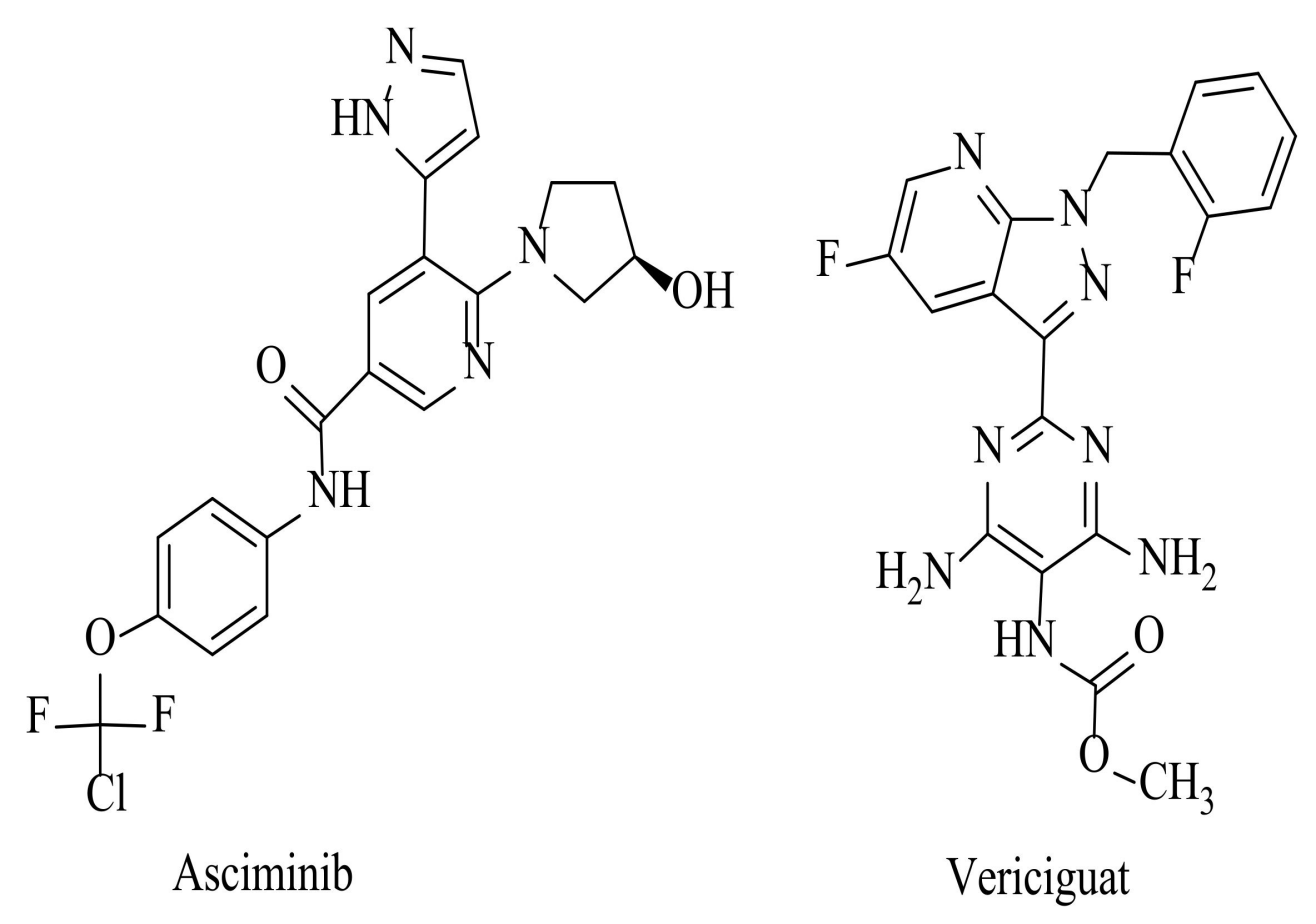
Structure of approved drug with pyrazolopyridine scaffold.

Furthermore, this scaffold is present in drugs approved by FDA in 2021, such as Asciminib, an allosteric inhibitor of BCR-ABL1 tyrosine kinase, and Vericiguat (Verquvo), a medication used to reduce the risk of cardiovascular death and heart failure (Figure 3).

Finally, we should mention the important role of sulfonamide derivatives, which are known to possess a wide range of activities, such as antimicrobial [40,41], anticancer [42,43], anti-inflammatory [44,45], antioxidant [44], antidiabetic [46], antimalarial [43], DHFR inhibitory [47], and carbonic anhydrise inhibitory [48,49] activities. Furthermore, they seem to play a significant role in carbonic anhydrise inhibition, since the sulfonamide group acts as a zinc binder [50].

The aim of this study is to support and extend our previous studies [51,52,53] on hCA as a target against diverse pathological conditions. Thus, herein we report the synthesis of two different groups of compounds, one of which is pyrazolo[4,3-c]pyridine sulfonamides (**1a**–**f**) and the other sulfonamide derivatives of different hetrocyclic moieties (**1g–1k**), and the evaluation of their inhibitory activities towards four human CAs (I, II, IX, and XII) as well as 3β and 3γ CAs from different bacterial strains.

## 2. Results and Discussion

### 2.1. Chemistry

**Scheme 1:**
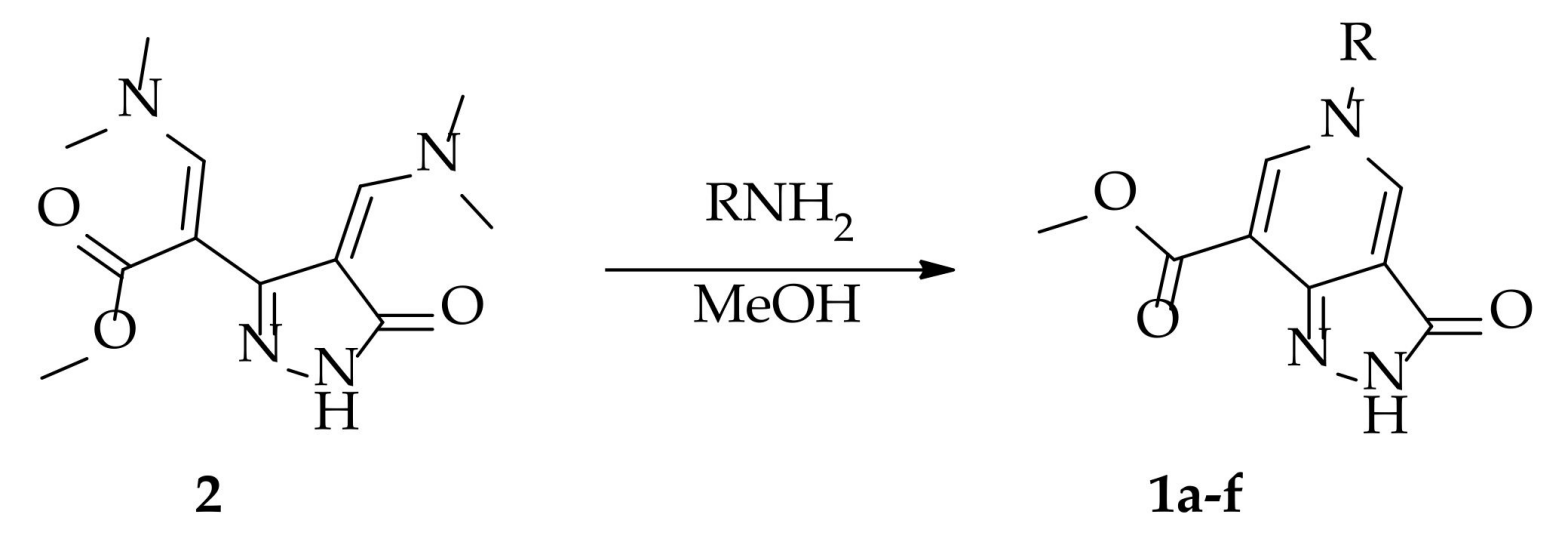
Synthesis of compounds **1a–f**.

The target pyrazolo[4,3-c]pyridines **1a–f** were obtained on the basis of dienamine **2**. Starting compound **2** was synthesized by the known two-step procedure from dimethyl acetonedicarboxylate [54]. The condensation of dienamine **2** with various amines containing sulfonamide fragments led to the final pyrazolo[4,3-c]pyridines **1a–f**. The reaction was carried out by reflux in methanol for 1 h, wherein the target products **1a–f** were obtained in 72–88% yields. This method allowed the synthesis of compounds **1a–f** containing various substituents at the nitrogen atom of pyridine moiety (Scheme 1, Table 1).

**Scheme 2:**
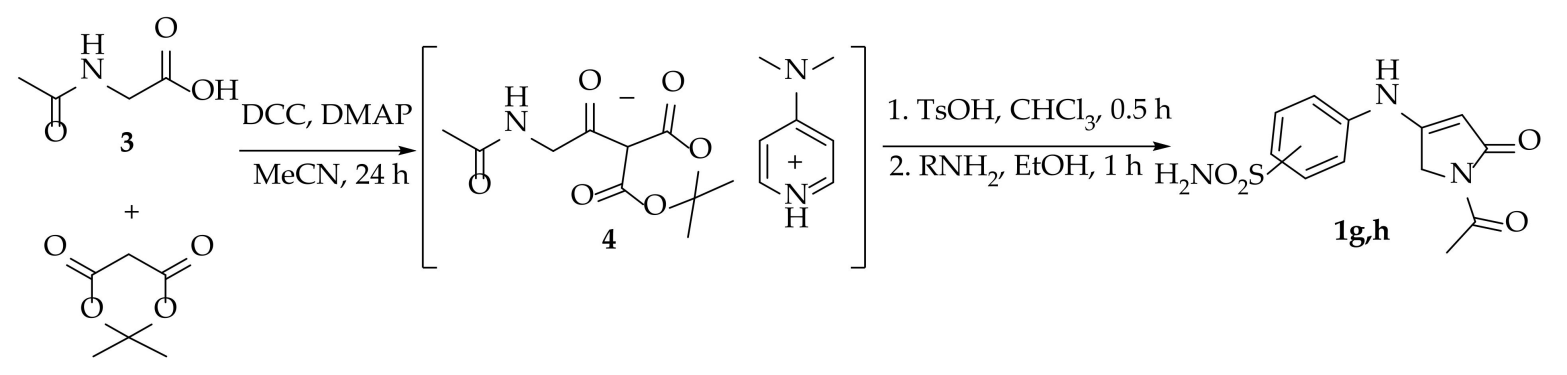
Synthesis of compounds **1g**,**h**.

*N*-Acetylpyrrol-2-ones **1g**,**h** were synthesized by a one-pot telescoped process from *N*-acetylglycine **3**, based on the protocols described in the literature [55,56]. The subsequent interaction of compound **3** with Meldrum’s acid in the presence of DMAP and DCC followed by the acid-catalyzed cyclization of the obtained salt **4** and the final condensation with the corresponding sulphanilamides led to the target pyrrolones **1g**,**h**. The obtained products were synthesized with yields of 47% and 58% (Scheme 2).

**Scheme 3:**
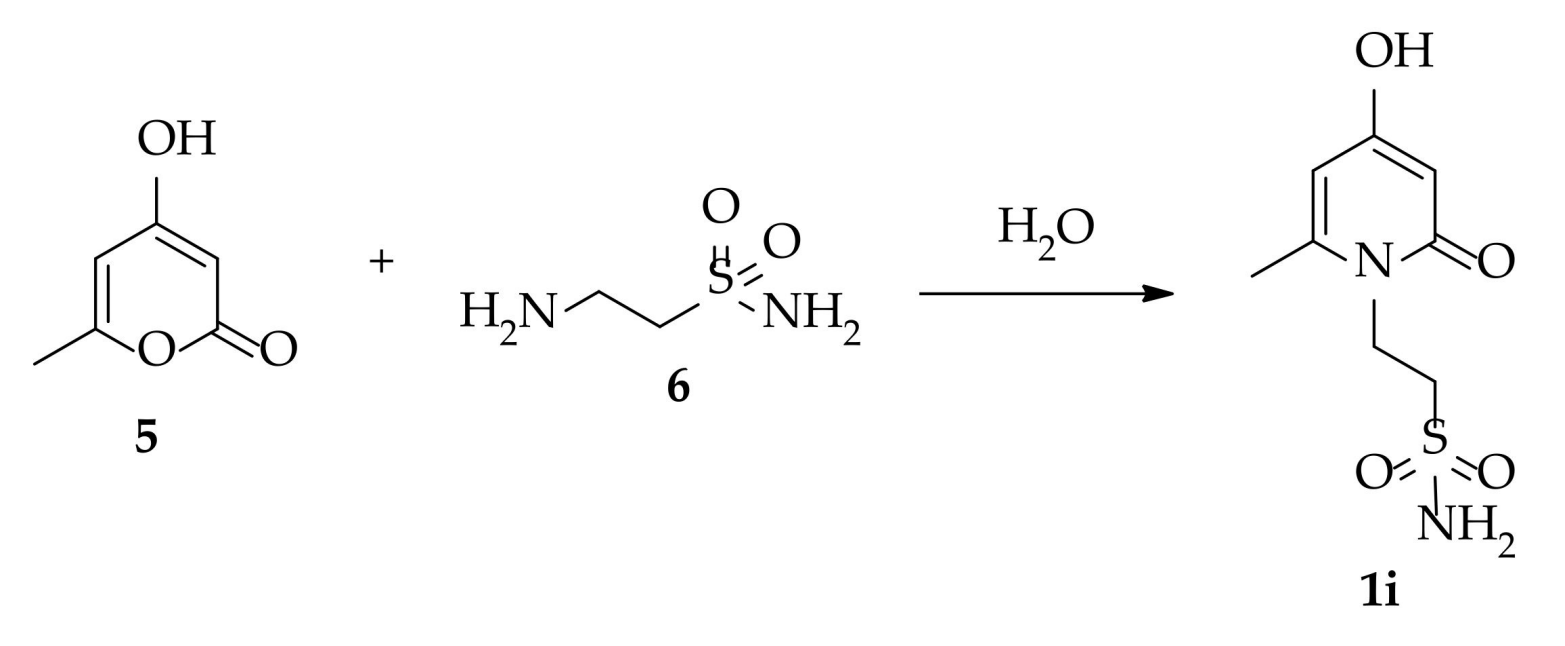
Synthesis of compound **1i**.

4-Hydroxypyridine-2-one **1i** was obtained by the reaction of 4-hydroxy-6-methyl-2-pyrone **5** with the corresponding sulfonamide **66** using the method described in the literature [57]. The process was carried out at reflux in water for 5 h, while the final product **1i** was synthesized with a 68% yield (Scheme 3).

**Scheme 4:**
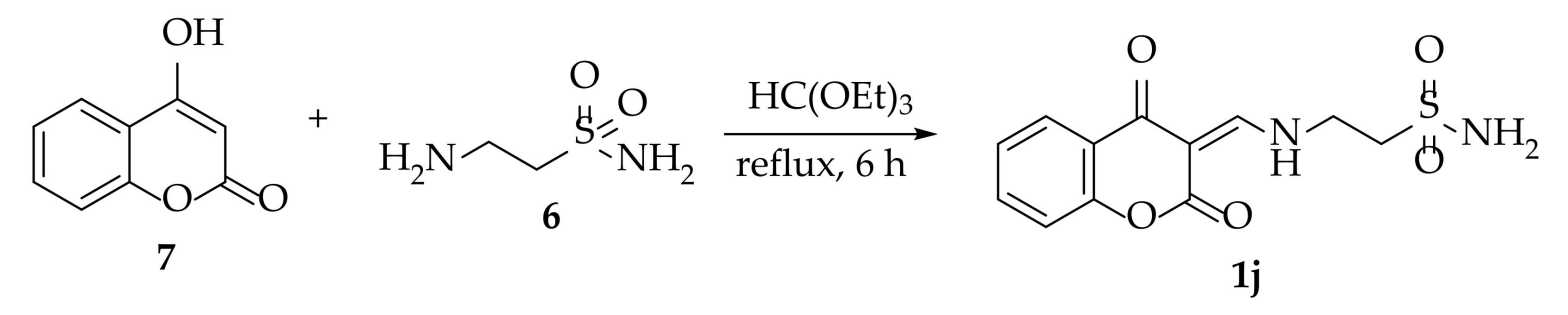
Synthesis of compound **1j**.

The target chromane-2,4-dione **1j** was synthesized by the condensation of 4-hydroxycoumarin **7** with sulfonamide **6** by the method described in the literature [58]. In the considered case, the excess of triethyl orthoformate was employed as a solvent, wherein the final product was obtained with a 47% yield (Scheme 4).

**Scheme 5:**
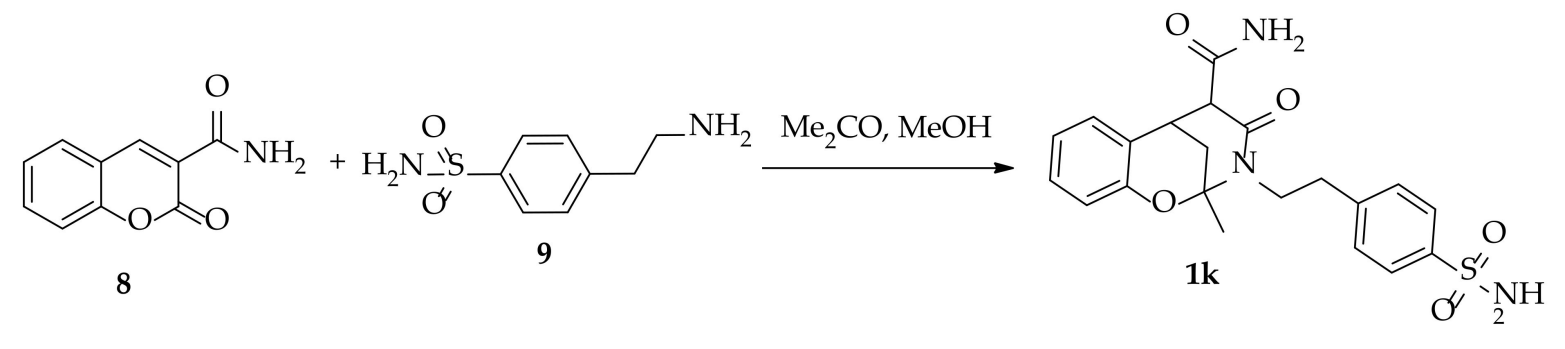
Synthesis of compound **1k**.

**Scheme 6:**
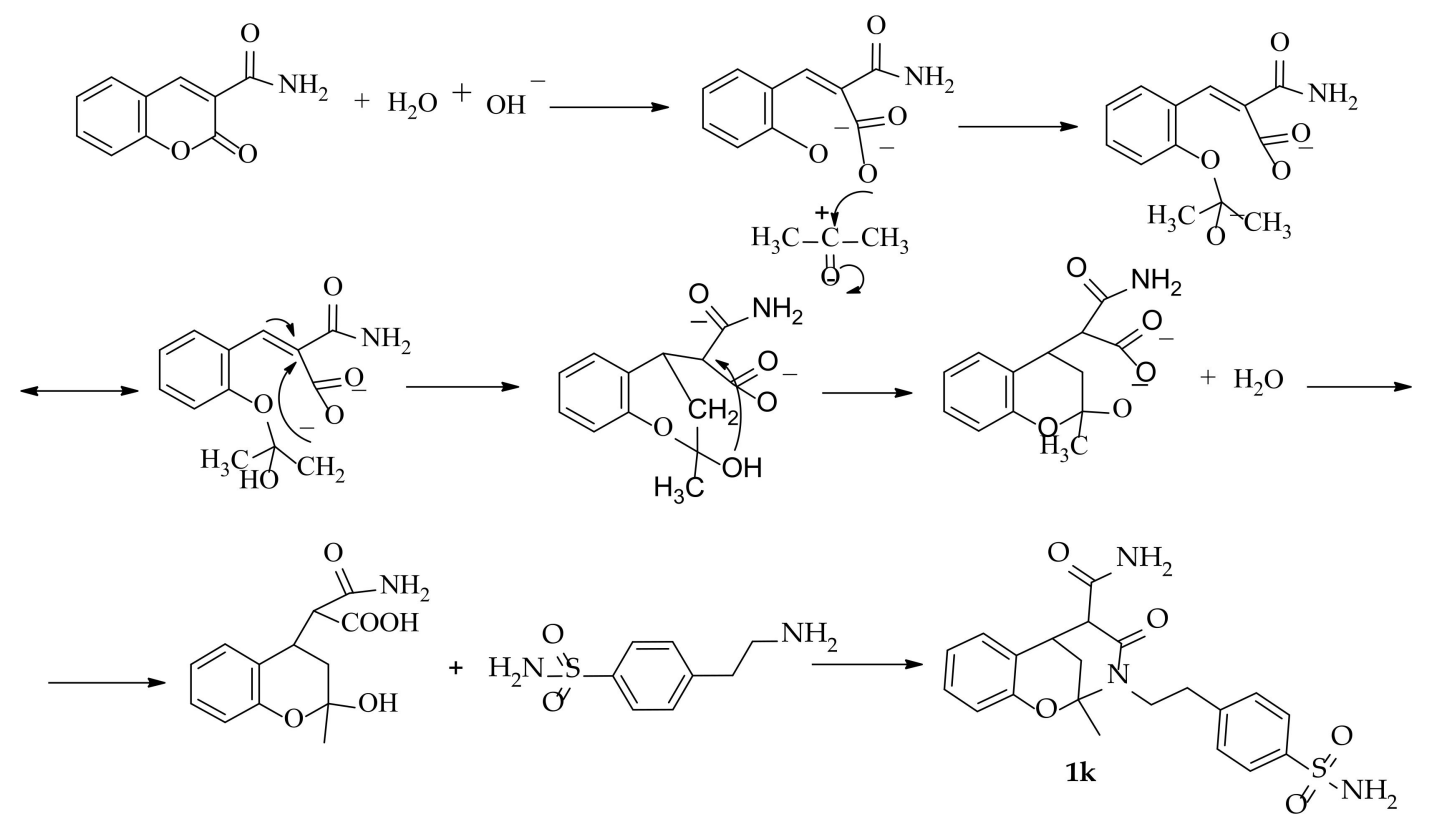
Probable mechanism of synthesis of **1k**.

Chromene-3-carboxamide **8** [2] was used as the starting compound for the synthesis of the target sulfonamide **1k** using the approach presented in the literature [59]. The interaction of compound **8** with amine **9** in the mixture of acetone and methanol at reflux resulted in the formation of the final product **1k** with a 56% yield (Scheme 5).The suggested mechanism of synthesis of compound **1k** is presented in Scheme 6.

The synthesized sulfonamides **1a–k** were solid crystalline compounds, whose structure was confirmed by ^1^H NMR spectroscopy. The ^1^H NMR spectra of the products contained characteristic signals of the protons of the sulfonamide moiety in the region δ 6.91–7.88 ppm. The remaining signals were also in good agreement with the presented structures. (The detail explanation is in the Supplementary Material).

### 2.2. Carbonic Anhydrase Inhibition

All the compounds (**1a–k**) were evaluated for their inhibitory activity against four human CA isoforms, namely, hCA I, hCA II, hCA IX, and hCA XII. The results are shown in Table 2.

From the data of Table 1, it is obvious that all the compounds inhibited all the human isoforms of CA used in this study, but with a varying range of inhibition constants. Thus, in the case of hCA I, the Ki values of the compounds ranged from 58.8 to 8010 nM. The best activity against the hCA I isoform was shown by compound **1f**, with a Ki of 58.8 nM, followed by **1g** and **1k** (Ki of 66.8 and 88.3 nM, respectively), being more potent than the reference drug acetazolamide (Ki = 250 nM). Compound **1a** exhibited the lowest activity. It should be mentioned that five out of the eleven compounds displayed higher activity against this isoform than AAZ.

The structure–activity relationship studies revealed that, in the group of pyrazolopyridine derivatives, the presence of an *N*-methylpropionamide linker between the benzensulfonamide and the methyl 3-oxo-3,5-dihydro-2H-pyrazolo[4,3-c]pyridine-7-carboxylate moiety (**1f**) is favorable for hCA I inhibitory activity. The straight connection between the two benzensulfonamides and the pyrazolopyridine moiety (**1b**) decreased activity against hCA I (~2.7 times), while the connection of the pyrazolopyridine moiety with the sulfonamide group through a CH_2-_CH_2_~linker (**1a**) was detrimental. On the other hand, the connection of the benzensulfonamide with the 1-acetyl-4-amino-1*H*-pyrrol-2(5*H-*one group by an NH group as a linker (**1g**) slightly decreased the activity compared to the compound **1f**, while the presence of a -3,4,5,6-tetrahydro-2*H*-2,6-methanobenzo[g][1,3]oxazocine ring connected to the benzensulfonamide through a CH_2_CH_2_ linker (**1k)** led to a slightly less active compound. The presence of 4-hydroxy-1,6-dimethylpyridin-2(1H)-one (**1i)** and chroman-2,4-dione (**1j**) moieties were not favorable for the activity against the hCA I isoform.

As far as the inhibition of the hCA II isoform is concerned, the Ki values of the tested compounds ranged from 5.6 to 7329 nM. The highest activity against the hCA II isoform was observed for compound **1k**, with a Ki value of 5.6 nM, followed by compound **1f** (KI = 6.6 nM), being more potent than the reference drug AAZ (Ki = 12.1). It should be mentioned that these two compounds were among the top-three most active against the hCA I isoform. Both compounds were very selective, with selectivity indexes (SIs) of 15.8 and 9 toward hCA I and 75.25 and 137.5 towards hCA IX, respectively, while the SIs towards hCA XII were 6 and 71.9 for **1k** and **1f**, respectively. The lowest activity was exhibited by compound **1j**, (E)-2-(2,4-dioxochroman-3-ylidene)ethanesulfonamide.

According to the structure–activity relationships, it is obvious that the presence of a 3,4,5,6-tetrahydro-2*H*-2,6-methanobenzo[g][1,3]oxazocine ring (**1k**) was beneficial for activity against the hCA II isoform. The replacement this ring by a pyrazolopyrimidine ring connected to the benzensulfonamide by an *N*-methylpropionamide linker (**1f**) slightly decreased the activity, while the removal of the *N*-methylpropionamide linker from compound **1f** led to a less active compound, **1b**. The presence of ethanesulfonamide (**1j**) instead of benzensulfonamide in compound **1f** and chroman-2,4-dione had a negative impact on the inhibition of the hCA II isoform.

None of the compounds exceeded the activity of the reference drug (Ki = 25.8 nM) against the hCA IX isoform. The compounds showed moderate-to-low activity against this isoform, with a Ki ranging from 79.6 nM to 907.5 nM. Nevertheless, compounds **1a** and **1i** were found to be very selective towards hCA I and hCA II, with SIs of 81.8, 74.9 for hCA I and 68.3 and 85.3 for hCA II, respectively.

Concerning the hCA XII isoform, although the compounds exhibited moderate-to-low activity against it, they were more potent than against the hCA IX isoform. The Ki values of the compounds against the hCA XII isoform were between 34.5 and 713.6 nM, compared to 5.7 nM for AAZ. Furthermore, compound **1k** was selective towards hCA IX, with an SI of 12.2.

The general conclusion is that these compounds appeared to be more potent against the hCA I isoform, while the two the most active compounds against the hCA II isoform were very selective. In the case of hCA I, the shifting of the 4-((1-acetyl-5-oxo-2,5-dihydro-1*H*-pyrrol-3-yl) amino) substituent on benzensulfonamide (**1g**) to position 3 decreased the activity slightly, while in the case of the hCA II and hCA XII isoforms, the activity decreased more, though the order of the activity remained the same. In the case of the hCA IX isoform, the 3-((1-acetyl-5-oxo-2,5-dihydro-1*H*-pyrrol-3-yl)amino) substituent was more beneficial than the 4-((1-acetyl-5-oxo-2,5-dihydro-1H-pyrrol-3-yl)amino) substituent.

In addition, we investigated the activity of our compounds towards three beta and three gamma CAs from different microorganisms (Table 3). It was found that the compounds showed inhibitory activity against all the bacterial CAs examined but to carrying extents. Thus, the activity of the compounds against the β-CA from *E. coli* was in the Ki range of 94.9 nM to 5027 nM, and only one compound (**1j**, Ki = 94.9 nM) was more active than AAZ (Ki 227 = nM). Much better activity was observed against the β-CA from *Burkholderia pseudomallei (BpsCAβ)*. The K_i_ values varied from 96.4 to 788.8 nM compared to AAZ (Ki = 745 nM). Thus, the best activity was achieved for compound **1i**, with a K_i_ value of 96.4 nM. Furthermore, this compound showed quite good selectivity against the β-CA from *E. coli* (SI 31.6) and the β-CA from *Vibrio cholerae (Vh*CAβ, SI 23.2*).* It should be mentioned that eight out of the eleven compounds appeared to be more potent than AAZ. On the other hand, only two compounds, **1k** and **1f**, displayed good activity against *Vh*CAβ, with K_i_ values of 355.8 and 466.6 nM, respectively, compared to AAZ (K_i_ = 451 nM).

The structure–activity relationships revealed that the presence of the 4-hydroxy-6-methylpyridin moiety (**1i**) was beneficial for the activity against *BpsCAβ,* while a positive influence on the activity against this isoform from *E. coli* was observed in the case of the presence of a chroman-2,4-dione scaffold (**1j**). The replacement of the 4-hydroxy-6-methylpyridin moiety with the 3,4,5,6-tetrahydro-2*H*-2,6-methanobenzo[g][1,3]oxazocine-5-carboxamide moiety (**1k**) decreased the activity slightly, but the compound still remained one of the most active against *BpsCAβ*. The introduction of pyrazolopyridine to the benzensulfonamide moiety via an *N*-methylpropionamide linker led to a less active compound, **1b** (though this was still one of the active compounds), while the presence of the acetyl aminopyrole moiety in position 3 of the benzensulfonamide had a very negative impact. On the other hand, the presence of a 3,4,5,6-tetrahydro-2*H*-2,6 -methanobenzo[g][1,3]oxazocine ring (**1k**) and pyrazolopyridine connected to benzensulfonamide by an *N*-methylpropionamide linker (**1f**) had a positive effect on the activity against the β-CA isoform from *VhCA.*

As far as the γ-CAs are concerned, the best activity was observed against this enzyme from *E. coli.* Ten out of eleven compounds were found to be more potent than AAZ (K_i_ = 248 nM). The best activity was shown by compound **1b**, followed by **1e**, with K_i_ values of 57.8 nM and 58.1 nM, respectively. These two compounds displayed almost the same selectivity towards the β-CA from *E. coli*, *Bps*CA*β, BpsCAγ, and the* γ-CA from *Porphyromonas gingivalis (PgiCAγ),* with SIs *of 59.8, 3.96, 8.88, and 1.59, respectively.* The tested compounds also expressed good activity against *PgiCA*γ, with K_i_ values in the range of 84.3–848 nM, compared to AAZ (K_i_ = 324 nM). The activity order was **1g** > **1j** > **1h** > **1b** > **1i** > **1e** > **1k** > **1c** > **1f** > **1d** > **1a**. Compound **1g** was the most active, followed by **1j**, with K_i_ values of 83.1 and 84.3 nM, respectively. It should be mentioned that compound **1g** showed selectivity (SI 17.4) towards *Vh*CA*β*. In the case of *PgiCA*γ, seven out of the eleven compounds showed better activity than AAZ. On the other hand, the compounds were less potent against *BpsCA*γ*:* only two compounds, **1g** and **1i** (with K_i_ values of 97.1 and 191.5 nM, respectively), appeared to be more potent than AAZ (Ki = 149 nM). The comparison of the activity of the compounds against the different β- and γ-CAs revealed that the tested compounds were more active against the γ-CA from *E. coli* than the β-CA, while the opposite was observed in the case of the enzymes originating from *BpsCA*.

According to the structure–activity relationships, the presence of pyrazolopyridine at position 4 of benzensulfonamide (**1b**) was favorable for the activity against the γ-CA isoform from *E. coli*. The introduction of an ethyl linker between these two moieties led to a slightly less active compound, **1e**, while the connection of the substituted pyrazolopyridine moiety directly to the sulfonamide group led to compound **1a**, with decreased activity. Nevertheless, all three of these compounds were among the most active against this isoform from *E. coli*, while the presence of the 1-acetyl-4-amino-1-pyrrol-2(5*H*)-one substituent (**1h**) at position 3 of benzensulfonamide was detrimental, as in the case of the inhibition of *BpsCA*β.

In the case of the inhibition of *PgiCA*γ, a positive influence was observed for the presence of the 1-acetyl-4-amino-1*H*-pyrrol-2(5*H*)-one substituent (**1g**) at position 4 of benzensulfonamide, followed by the (E)-3-(aminomethylene)chroman-2,4-dione (**1e**) substituent. The replacement of the two previous substituents by the 1-acetyl-4-amino-1-pyrrol-2(5*H*)-one substituent (**1h**) decreased the activity compared to compound **1e**. However, compounds **1e** and **1h** were among the most active. Finally, the presence of the methyl 3-oxo-5-propyl-3,5-dihydro-2*H-*pyrazolo[4,3-c]pyridine-7-carboxylate moiety directly connected to the sulfonamide group had a negative effect on the activity against this isoform. Regarding *BpsCA*γ, the most beneficial impact on the activity against this isoform appeared to come from the presence of the 1-acetyl-4-amino-1*H*-pyrrol-2(5H)-one substituent (**1g**) at position 4 of benzensulfonamide, as well as the 4-hydroxy-6-methylpyridin-2(1*H*)-one moiety (**1i**) connected to the sulfonamide group via ethylene, while this linker between the 3-ethyl-2-methyl-4-oxo-3,4,5,6-tetrahydro-2*H*-2,6-methanobenzo[g][1,3]oxazocine-5-carboxamide (**1k**) moiety and benzensulfonamide was unfavorable.

It should be mentioned that compound **1b** was selective against *E. coli* β, with a selectivity index (SI) of 59.8; BpsCA**β** (SI 3.96); BpsCAγ (SI 8.88); *Pgi*CAγ (SI 1.57); and *Vh*CAβ (SI 14.6).

### 2.3. Molecular Docking Studies

#### 2.3.1. Molecular Docking Studies in Human CA Isoforms

For the docking studies, the most active compounds (**1c**, **1g**, **1f** and **1k**) were selected to be studied as representative of the whole set of compounds, in order to predict the possible mechanism of inhibition. 

It is known that all human CA isoforms have an analogous active site containing His94, His96, and His119 as conserved residues. These residues act as zinc ligands. Additionally, the active site of all isoforms contains two other conserved residues, Thr199 and Glu105, acting as “gate keepers” [60,61,62,63]. However, these isoforms differ mostly in the residues in the middle and at the exit of the active site cavity.

The results of the molecular docking studies of the tested compounds on the hCA I, II, IX, and XII isoforms are presented in Table 4. According to these results, all tested compounds bind the enzymes in the same manner, chelating the Zn (II) ion in a deprotonated form as anions (negative nitrogen of the sulfonamide group) [63]. 

The docking results showed that the selectivity profile as well as the inhibition mode of some compounds to each isoform depend on the variances in the active sites of the enzymes. In particular, the conformation that the compounds adopt within the enzyme active site and their interactions are affected by the nature of the amino acids of the active site of each enzyme.

**Figure 4:**
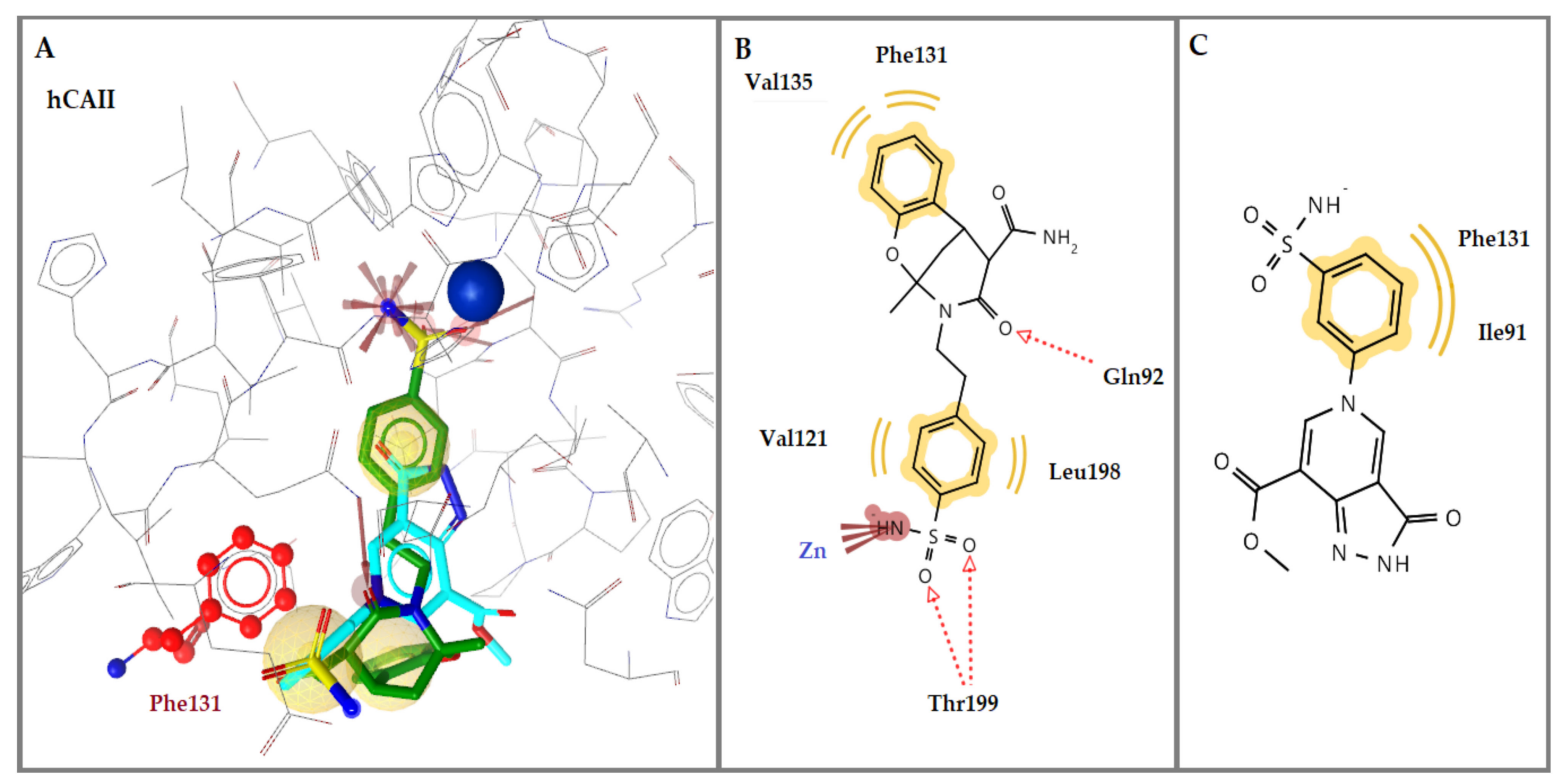
(**A**) Superposition of compound **1k** (green) bound to hCA II in comparison with compound **1c** (light blue) bound to hCA II, with specific residues labeled. (**B**) 2D interaction diagram of compound **1k** docking pose interactions with the key amino acids in hCA II. (**C**) 2D interaction diagram of compound **1c** docking pose interactions with the key amino acids in hCA II. Active-site zinc shown as blue sphere, red dotted arrows indicate H-bond, and yellow spheres are hydrophobic interactions.

Taking all this into account, comparing the docking poses in the hCA II enzyme of compounds **1k** and **1c**, with Ki values for the hCA II enzyme of 5.6 nM and 247.4 nM, respectively, we can say that the presence of a longer ethyl chain in compound **1k** plays an important role in the inhibition profile of this compound compared to compound **1c**. The hCA II enzyme has a hydrophobic residue Phe131 in the active site that provides a bulky environment for the compound to freely enter the active site. The longer ethyl chain of compound **1k** gives it flexibility and enables it to avoid the steric hindrance of the bulky residue Phe131 in the hCA II isoform, increasing the inhibition potency (Figure 4). 

As is illustrated in Figure 4, this compound inserts itself into the active site of the enzyme freely, and the negative nitrogen of the sulfonamide group chelates the Zn (II) ion and forms hydrogen bonds. Moreover, the oxygen atoms of the sulfonamide group form hydrogen bonds with residue Thr199 (distance 2.54 and 1.98, respectively) and the oxygen atom of the carbonyl group of the compound forms another H-bond with residue Gln92 (distance 2.45). Furthermore, the benzene moiety interacts hydrophobically with residues Val121 and Leu198. These interactions further stabilize the complex and explain its high inhibition potency (Figure 4B).

On the other hand, compound **1c**, probably because of the presence of the bulky Phe131 residue in the hCA II enzyme and in accordance with its bulky and unbent structure, cannot enter the active site of the enzyme, resulting in its low inhibition potency (Figure 4A,C).

**Figure 5:**
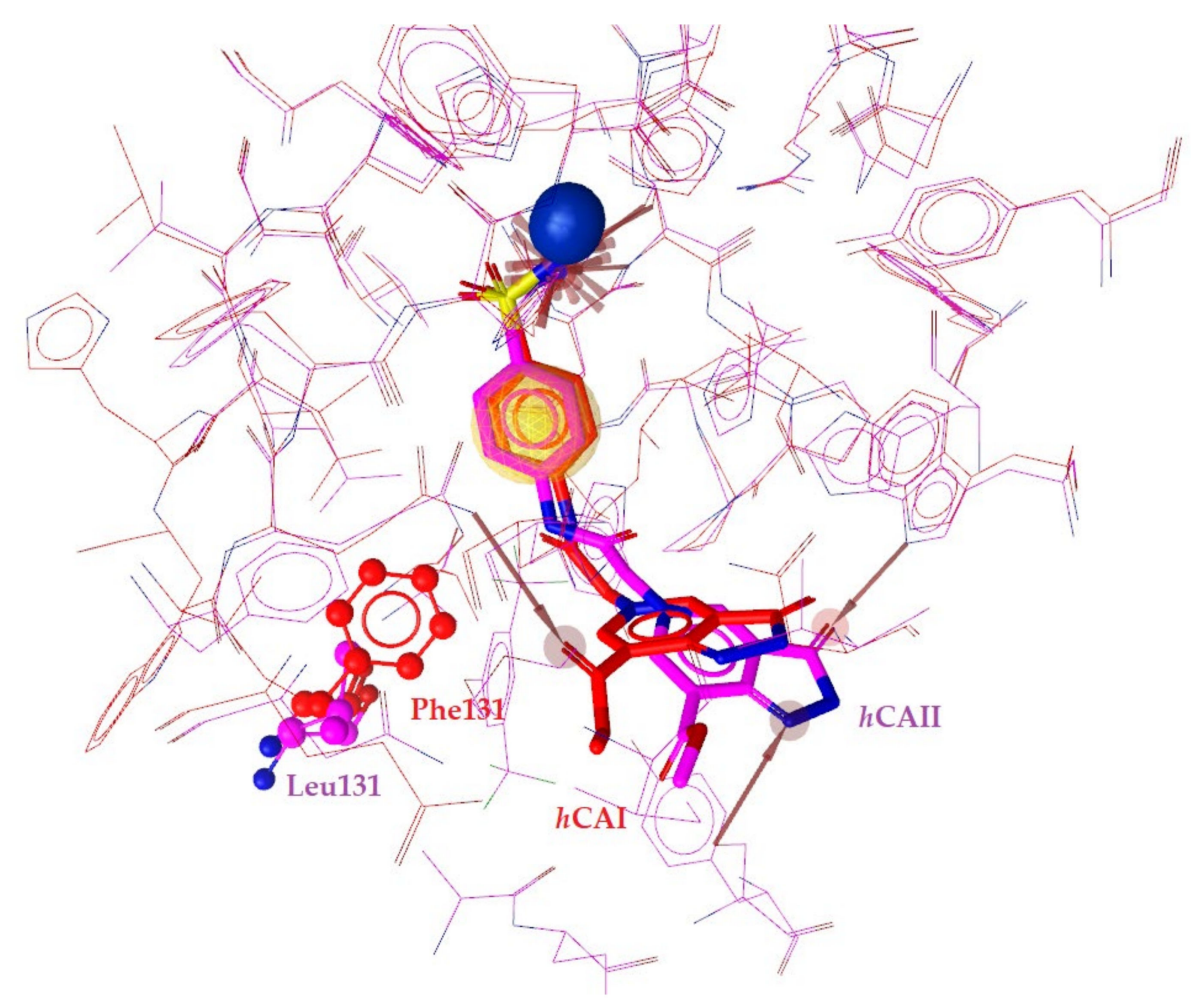
Superposition of compound **1f** bound to hCA I (red) in comparison to hCA II (magenta), with specific residues labeled. Active-site zinc shown as blue sphere, red dotted and green arrows indicate H-bonds, and yellow spheres are hydrophobic interactions.

The flexible structure of compound **1f** can also explain its inhibition potency towards the hCA II and hCA I enzymes, with Ki values of 6.6 nM and 58.8 nM, respectively. Indeed, the superposition of this compound bound to hCA I in comparison to hCA II (Figure 5) shows that it can adopt a conformation that favors the interaction with the active sites of both the isoforms, avoiding the steric hindrance of the bulky residue Phe131 in the hCA II isoform and increasing the stability of each complex and subsequently the inhibition potency of the compound.

**Figure 6:**
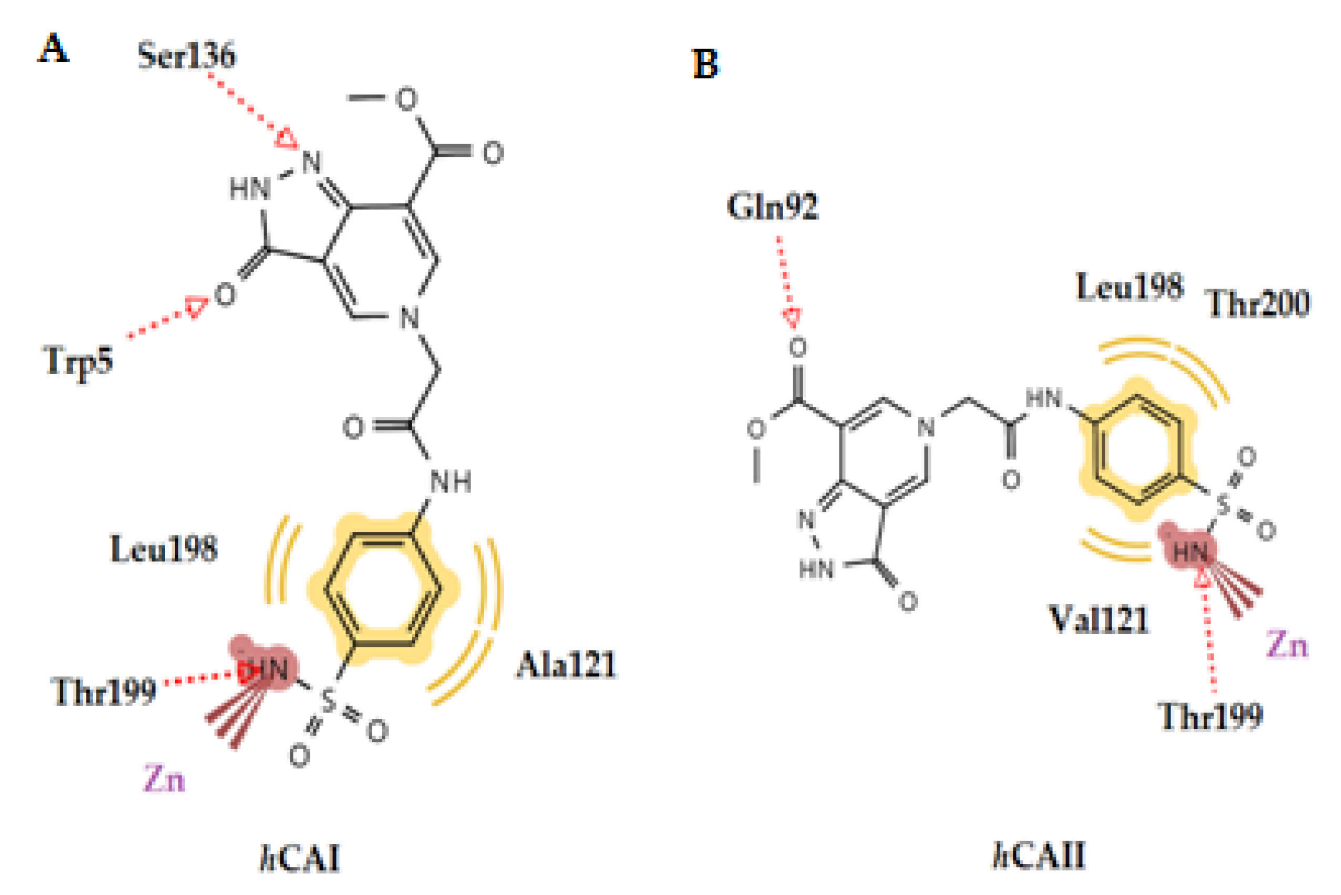
2D interaction diagram of compound **1f** docking pose interactions with the key amino acids in (**A**) hCA I and (**B**) hCA II.

In particular, in both structures, the negative nitrogen of the sulfonamide group chelates the Zn (II) ion and forms hydrogen bonds (Figure 6A,B). In both isoforms, the single oxygen atom of the sulfonamide group forms a hydrogen bond with residue Thr199. Moreover, in the isoform hCA I, the N atom of the heterocycle ring forms another H-bond with residue Ser135, as well as between the oxygen atom of the carbonyl group of the compound and residue Trp5. On the other hand, the benzene ring interacts hydrophobically with Val121 and Leu198 (Figure 6A,B). These interactions can probably explain the high Ki value of compound **1f** against hCA II and the other isoforms.

**Figure 7:**
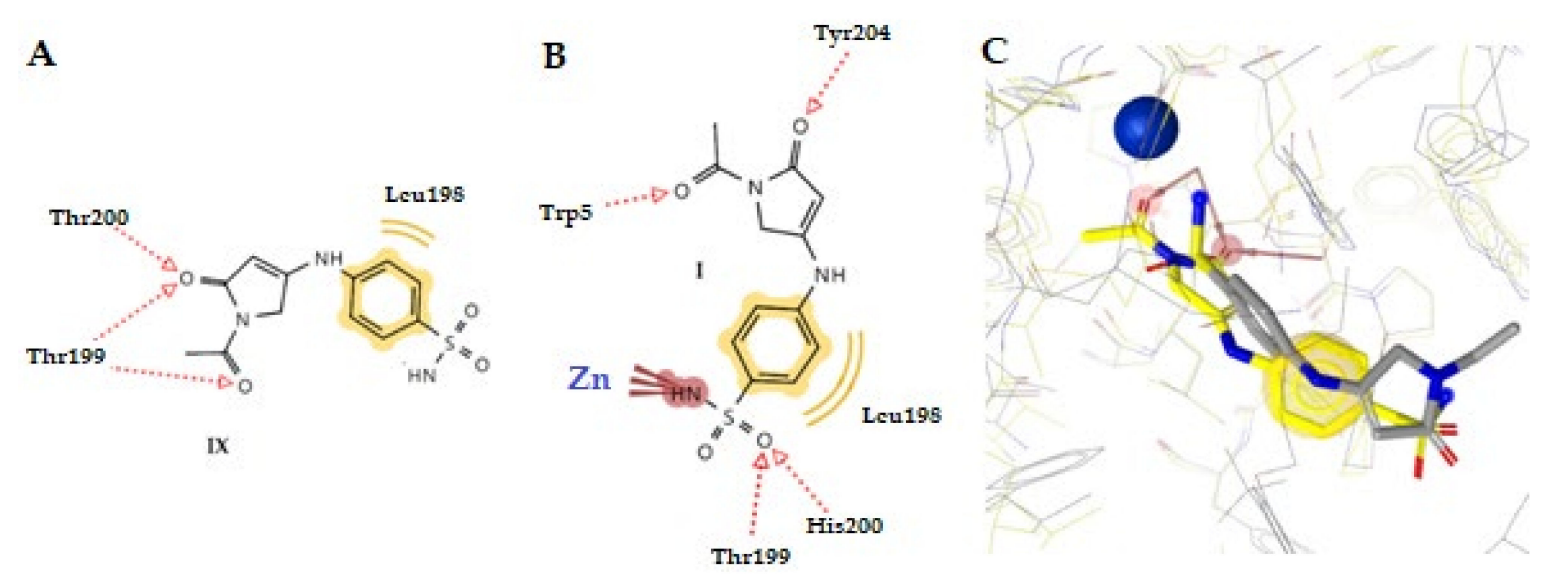
2D interaction diagram of compound **1g** docking pose interactions with the key amino acids in (**A**) hCA IX and (**B**) hCA I. (**C**) Superposition of compound **1g** bound to hCA I (yellow) in comparison to hCA IX (grey). Active-site zinc shown as blue sphere, red dotted arrows indicate H-bonds, and yellow spheres are hydrophobic interactions.

Finally, the docking pose of compound **1g** in the active site of the hCA I isoform can reveal the probable reason of its high inhibition profile (Ki = 66.8 nM). As is illustrated in Figure 7, compound **1g** binds hCA I with the carbonyl substituent forming a hydrogen bond with residue Trp5 and the carbonyl group of the heterocyclic ring with Trp204, respectively. The comparison of the two binding modes of the compound in the hCA I and hCA IX isoforms revealed that, while compound **1g** binds in the hCA I isoform with the negative nitrogen of the sulfonamide group, chelating the Zn (II) ion, in the hCA IX isoform this interaction is not present. One reason may be the fact that because of the large size of the active site of the hCA IX isoform, this compound interacts with residues forming hydrogen bonds that do not let it reach the Zn ion and interact with it. This is probably the reason why compound **1g** has such a low inhibition against the hCA IX isoform (Ki value of 294.2 nM).

#### 2.3.2. Molecular Docking Studies in β- and γ-CA Classes

The CA enzymes of bacteria belong to three known classes (α, β, and γ) [64,65]. The α- and β-CAs use the Zn(II) ion as a catalytic metal, while γ-CAs are Fe(II) enzymes which also actively bind Zn(II) or Co(II) ions [66]. In the α- and γ-classes, three His residues from the CA active site are coordinated with the metal ion; in the β-class, one His and two Cys residues. Moreover, an incoming water molecule approaches the metal ion (as a hydroxide ion), which is responsible for the catalytic activity [67]. 

X-ray crystal structures are available for several β-CAs, such as those from *Escherichia coli*, *Mycobacterium tuberculosis,* and *Vibrio cholerae* [67]. For this study, we used the structure of the *E. coli* β-carbonic anhydrase (PDB code: 1IP6) in order to examine the way our compounds interact.

As the only enzyme crystalized so far from the γ-class of carbonic anhydrases is CAM (Carbonic Anhydrase *Methanosarcina*) from *Methanosarcina thermophila* [68], we used this enzyme for the docking studies. This enzyme contains a glutamic acid residue (Glu89) instead of a histidine (as in α-CAs), acting as a proton shuttle residue.

The results of the docking studies of the tested compounds in both enzymes are presented in Table 5.

**Figure 8:**
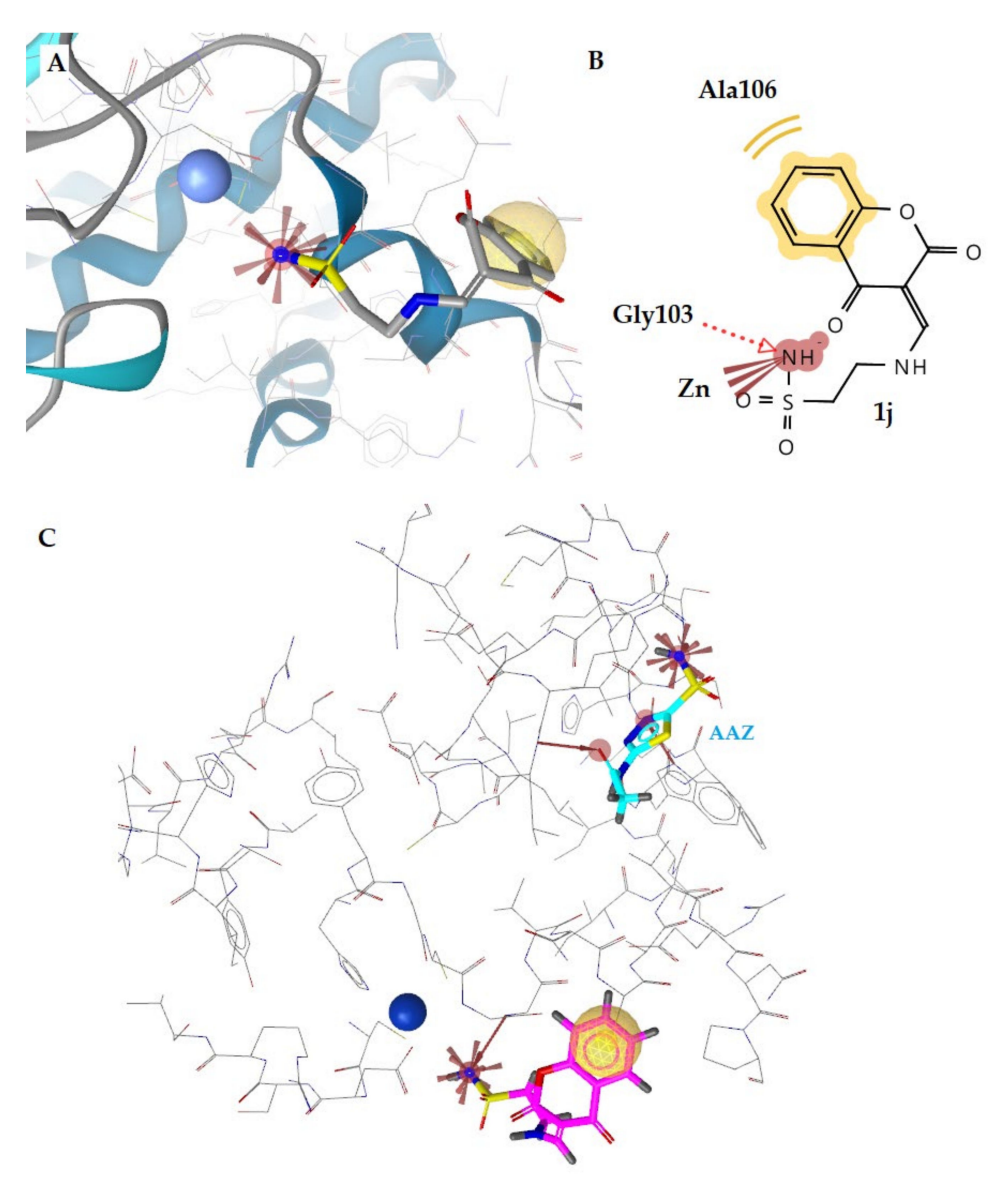
(**A**) Docking pose of compound **1j** in *E. coli* β-CA enzyme. (**B**) 2D interaction diagram of compound **1j**. (**C**) Superposition of compound **1j** bound to *E. coli* β-CA enzyme (magenta) in comparison to AAZ (blue). Active-site zinc shown as blue sphere, red dotted arrows indicate H-bonds, and yellow spheres are hydrophobic interactions.

Compound **1j**, with the best inhibition profile for the *E. coli* β enzyme, seemed to interact with the active site of the enzyme, chelating the Zn ion. Moreover, the hydrophobic interactions and the formation of a hydrogen bond between the N atom of the NH2 group and residue Gly103 provide stability to the enzyme–compound complex (Figure 8A,B). On the other hand, the reference drug AAZ seemed to bind in a cavity away from the active site of the enzyme, and this may be the reason for its low K_i_ value (227 nM) (Figure 8C).

**Figure 9:**
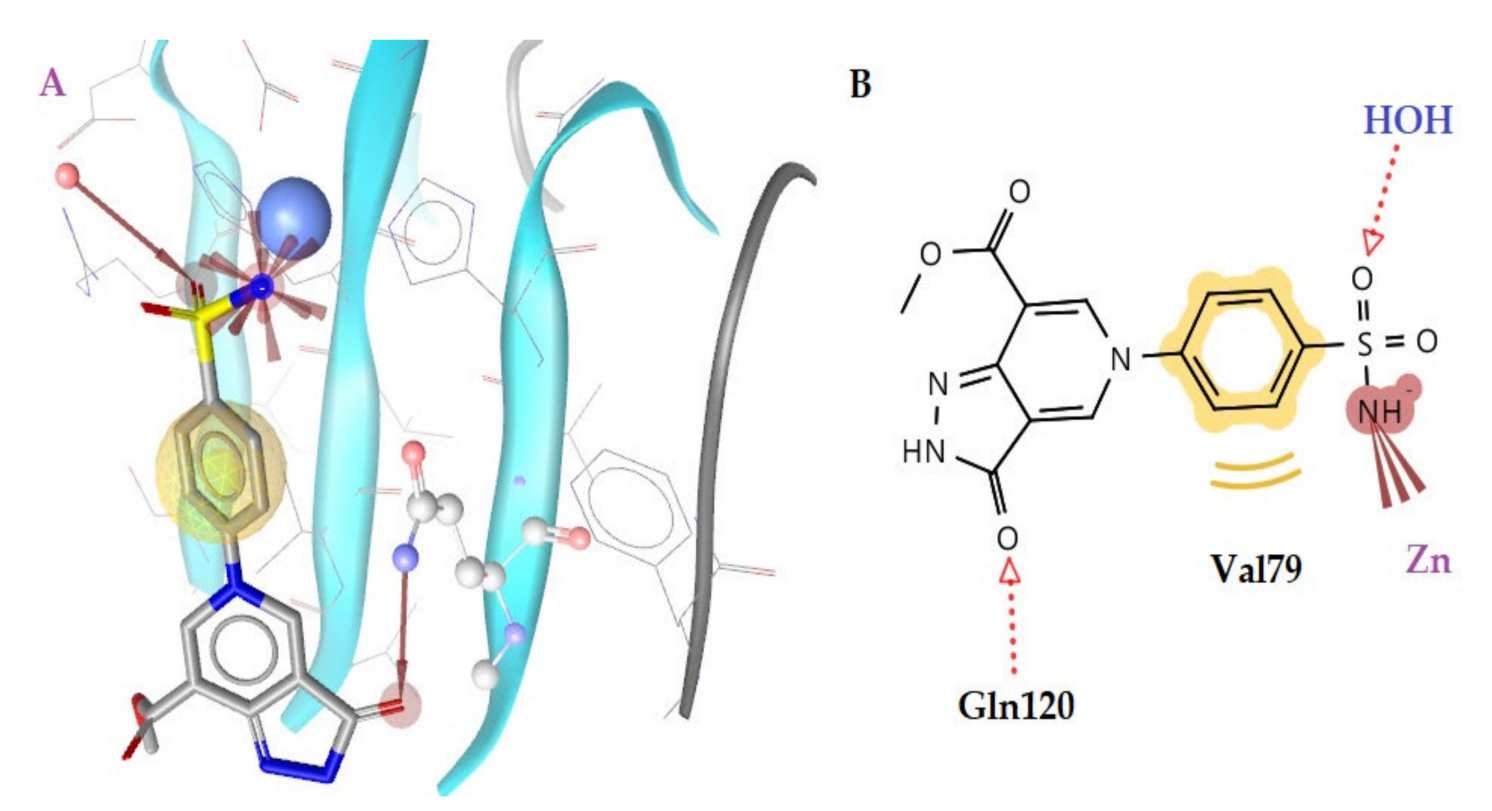
(**A**) Docking pose of compound **1b** in γ-CA enzyme. (**B**) 2D interaction diagram of compound 1b. Active site zinc shown as blue sphere, water molecule shown as red sphere, red dotted arrows indicate H-bonds, and yellow spheres hydrophobic are interactions.

The high inhibition profile of the compounds against the γ-CAs can be attributed to their ability to adopt a conformation inside the active site of the enzyme interacting with both the zinc ion and the water molecule responsible for the catalytic activity of the enzyme. This phenomenon was observed in particular for the most-active compounds, **1b**, **1e**, **1j**, and **1k**. All of these compounds interacted by forming a hydrogen bond between the oxygen atom of the sulfonamide group and the water molecule (Figure 9), explaining their high inhibition profile.

### 2.4. In Silico Prediction Studies

#### Drug-Likeness

Drug-likeness was examined as a significant tool for the prediction of whether the molecules could be a powerful drug candidate. Several rules, such as those described by Lipinski [69], were used, and the bioavailability and drug-likeness scores are given in Table 5.

According to the prediction results, the bioavailability score of all compounds was about 0.55. Furthermore, all compounds displayed good drug-likeness scores, ranging from −0.94 to 0.90. The best scores in the in silico prediction results were achieved by the most active compounds (**1f**, **1g** and **1k**), with drug-likeness scores of 0.93, 0.90, and 0.44, respectively (Table 6). Moreover, these compounds showed no rule violation, except compound **1f**, with one violation in Lipinski’s rule. From the table, it is obvious that only compounds **1g**–**1i** can be orally absorbed (TPSA 105.92–117.44), since TPSA values over 120 Ang^2^ are not favorable for oral absorption.

## 3. Materials and Methods

### 3.1. Chemistry

Unless otherwise stated, all starting chemicals were commercially available and were used as received. NMR spectra were recorded with Bruker AM 300 (300 MHz) and Bruker AV 400 (400 MHz) spectrometers in DMSO-*d*_6_. Chemical shifts (ppm) are given relative to solvent signals (DMSO-*d*_6_: 2.50 ppm (^1^H NMR)). The melting points were determined on a Kofler hot stage. 

#### 3.1.1. Synthesis of 5-Substituted Methyl 3-Oxo-3,5-dihydro-2H-pyrazolo[4,3-c]pyridine-7-carboxylates **1a–f** (General Procedure)

Mixture of dienamine 2 (0.53 g, 2 mmol) and corresponding amine (2.1 mmol) (0.22 g, 2.2 mmol of Et_3_N added in the case of amine hydrochloride) was refluxed in methanol (6 mL) for 1 h. The precipitate formed was collected by filtration, washed with methanol (3 × 5 mL), and dried to afford pure compounds **1a**–**f**.

Methyl 3-oxo-5-(2-sulfamoylethyl)-3,5-dihydro-2H-pyrazolo[4,3-c]pyridine-7-carboxylate **1a**: Yield 72%, m.p. > 300 °C. ^1^H NMR (300 MHz, DMSO-d_6_) δ 11.28 (br. s, 1H, NH); 8.51 (s, 1H, CH); 8.18 (s, 1H, CH); 6.95 (br. s, 2H, NH_2_); 4.49 (t, *J* = 6.3 Hz, 2H, CH_2_); 3.89 (s, 3H, OCH_3_); 3.55 (t, *J* = 6.3 Hz, 2H, CH_2_). ^13^C NMR (126 MHz, dmso) δ 164.45, 163.47, 141.64, 140.41, 139.98, 115.52, 111.75, 54.30, 52.39, 51.77, 39.68. Anal. Calcd. for C_10_H_12_N_4_O_5_S (%)—C, 40.00; H, 4.03; N, 18.66; O, 26.64; S, 10.68. Found (%)—C, 39.90; H, 4.01; N, 18.46.

Methyl 3-oxo-5-(4-sulfamoylphenyl)-3,5-dihydro-2H-pyrazolo[4,3-c]pyridine-7-carboxylate **1b**: Yield 88%, m.p. > 300 °C. ^1^H NMR (300 MHz, DMSO-*d*_6_) δ 11.47 (br. s, 1H, NH); 8.66 (s, 1H, CH); 8.20 (s, 1H, CH); 8.04 (d, *J* = 8.7 Hz, 2H, 2CH); 7.89 (d, *J* = 8.7 Hz, 2H, 2CH); 7.35 (br. s, 2H, NH_2_); 3.91 (s, 3H, OCH_3_). ^13^C NMR (300 MHz, DMSO) δ 164.21, 163.80, 145.91, 143.18, 139.78, 139.60, 138.43, 131.30, 127.55, 125.88, 121.02, 116.80, 112.63, 52.63. Anal. Calcd. for C_14_H_12_N_4_O_5_S (%)—C, 48.27; H, 3.47; N, 16.08. Found (%)—C, 48.21; H, 3.49; N, 15.96.

Methyl 3-oxo-5-(3-sulfamoylphenyl)-3,5-dihydro-2H-pyrazolo[4,3-c]pyridine-7-carboxylate **1c**: Yield 82%, m.p. >300 °C. ^1^H NMR (300 MHz, DMSO-*d*_6_) δ 11.49 (br. s, 1H, NH); 8.63 (s, 1H, CH); 8.20 (s, 1H, CH); 8.08 (s, 1H, CH); 7.94–7.86 (m, 2H, 2CH); 7.79–7.76 (m, 1H, CH); 7.28 (br. s, 2H, NH_2_); 3.88 (s, 3H, OCH_3_). ^13^C NMR (300 MHz, dmso) δ 164.20, 163.80, 145.91, 143.18, 139.88, 139.60, 138.43, 131.30, 127.55, 125.88, 121.32, 116.80, 112.63, 52.64. Anal. Calcd. for C_14_H_12_N_4_O_5_S (%)_—_C, 48.27; H, 3.47; N, 16.08. Found (%)—C, 48.15; H, 3.42; N, 16.15.

3-oxo-5-(4-sulfamoylbenzyl)-3,5-dihydro-2H-pyrazolo[4,3-c]pyridine-7-carboxylate **1d**: Yield 77%, m.p. > 300 °C. ^1^H NMR (300 MHz, DMSO-*d*_6_) δ 11.35 (br. s, 1H, NH); 8.61 (s, 1H, CH); 8.20 (s, 1H, CH); 7.86 (d, *J* = 8.5 Hz, 2H, 2CH); 7.58 (d, *J* = 8.5 Hz, 2H, 2CH); 7.19 (br. s, 2H, NH_2_); 5.41 (s, 2H, CH_2_); 3.87 (s, 3H, OCH_3_). ^13^C NMR (300 MHz, DMSO-d6) δ 164.31, 163.43, 144.46, 141.20, 140.47, 140.21, 139.55, 128.81, 126.77, 116.14, 112.40, 58.78, 52.47. Anal. Calcd. for C_15_H_14_N_4_O_5_S (%)—C, 49.72; H, 3.89; N, 15.46. Found (%)—C, 49.68; H, 3.95; N, 15.41.

Methyl 3-oxo-5-(4-sulfamoylphenethyl)-3,5-dihydro-2H-pyrazolo[4,3-c]pyridine-7-carboxylate **1e**: Yield 74%, m.p. > 300 °C. ^1^H NMR (300 MHz, DMSO-*d*_6_) δ 11.29 (br. s, 1H, NH); 8.53 (s, 1H, CH); 8.18 (s, 1H, CH); 7.78 (d, *J* = 8.6 Hz, 2H, 2CH); 7.49 (d, *J* = 8.6 Hz, 2H, 2CH); 7.11 (br. s, 2H, NH_2_); 4.35 (t, *J* = 6.2 Hz, 2H, CH_2_); 3.89 (s, 3H, OCH_3_); 3.20 (t, *J* = 6.2 Hz, 2H, CH_2_). ^13^C NMR (300 MHz, DMSO-d6) δ 164.38, 163.41, 143.04, 141.77, 141.14, 140.42, 139.64, 129.99, 126.22, 115.70, 111.85, 57.36, 56.48, 52.37, 36.51. Anal. Calcd. for C_16_H_16_N_4_O_5_S (%)—C, 51.06; H, 4.28; N, 14.89. Found (%)—C, 51.04; H, 4.15; N, 14.93.

3-oxo-5-(2-oxo-2-((4-sulfamoylphenyl)amino)ethyl)-3,5-dihydro-2H-pyrazolo[4,3-c]pyridine-7-carboxylate **1f**: Yield 85%, m.p. > 300 °C. ^1^H NMR (300 MHz, DMSO-*d*_6_) δ 11.31 (br. s, 1H, NH); 10.62 (br. s, 1H, NH); 8.42 (s, 1H, CH); 8.14 (s, 1H, CH); 7.81–7.72 (m, 4H, 4CH); 7.11 (br. s, 2H, NH_2_); 5.09 (s, 2H, CH_2_); 3.89 (s, 3H, OCH_3_). ^13^C NMR (300 MHz, DMSO-d6) δ 166.49, 164.45, 163.53, 142.43, 141.82, 141.27, 140.35, 139.17, 127.31, 119.19, 115.45, 111.30, 58.60, 52.35. Anal. Calcd. for C_16_H_15_N_5_O_6_S (%)—C, 47.40; H, 3.73; N, 17.28. Found (%)—C, 47.38; H, 3.79; N, 17.25.

#### 3.1.2. Synthesis of 1-Acetyl-4-(arylamino)-1,5-dihydro-2H-pyrrol-2-ones **1g**,**h** (General Procedure)

Mixture of *N*-acetylglycine 3 (0.47 g, 4 mmol), DCC (1.03 g, 5 mmol), DMAP (0.73 g, 6 mmol), and Meldrum’s acid (0.6 g, 4.2 mmol) in MeCN (10 mL) was kept for 24 h at room temperature. Then, solvent was evaporated, water (10 mL) was added to the obtained residue, and the reaction mixture was filtered from insoluble byproducts. Next, water solution was evaporated, TsOH hydrate (1.14 g, 6 mmol) in CHCl_3_ (10 mL) was added to the residue, and the obtained solution was refluxed for 0.5 h. Then, reaction mass was evaporated, corresponding sulphanilamide (4 mmol) in EtOH (10 mL) was added, and the mixture was refluxed for 1 h. Finally, the precipitate formed was collected by filtration, washed with ethanol (3 × 5 mL), and dried to afford pure compounds **1g**,**h**. 

4-((1-Acetyl-5-oxo-2,5-dihydro-1H-pyrrol-3-yl)amino)benzenesulfonamide **1g**: Yield 47%, m.p. 285–287 °C. ^1^H NMR (300 MHz, DMSO-*d*_6_) δ 9.81 (br. s, 1H, NH); 7.80 (d, *J* = 8.5 Hz, 2H, 2CH); 7.31 (d, *J* = 8.5 Hz, 2H, 2CH); 7.09 (br. s, 2H, NH_2_); 5.48 (s, 1H, CH); 4.38 (s, 2H, CH_2_); 2.40 (s, 3H, CH_3_). ^13^C NMR (300 MHz, DMSO-d6) δ 171.14, 168.63, 157.22, 143.48, 138.34, 127.82, 118.58, 92.97, 49.39, 24.36. Anal. Calcd. for C_12_H_13_N_3_O_4_S (%)—C, 48.81; H, 4.44; N, 14.23. Found (%)—C, 48.77; H, 4.48; N, 14.15.

3-((1-Acetyl-5-oxo-2,5-dihydro-1H-pyrrol-3-yl)amino)benzenesulfonamide **1h**: Yield 58%, m.p. 264–266 °C. ^1^H NMR (300 MHz, DMSO-*d*_6_) δ 9.80 (br. s, 1H, NH); 7.65 (s, 1H, CH); 7.55–7.45 (m, 2H, 2CH); 7.35–7.30 (m, 1H, CH); 7.21 (br. s, 2H, NH_2_); 5.49 (s, 1H, CH); 4.38 (s, 2H, CH_2_); 2.40 (s, 3H, CH_3_). ^13^C NMR (300 MHz, DMSO-d6) δ 171.21, 168.61, 157.69, 145.65, 141.05, 130.72, 122.41, 120.43, 115.67, 91.92, 91.90, 49.26, 40.03, 24.36, 24.33. Anal. Calcd. for C_12_H_13_N_3_O_4_S (%)—C, 48.81; H, 4.44; N, 14.23. Found (%)—C, 48.80; H, 4.53; N, 14.16.

#### 3.1.3. Synthesis of 2-(4-Hydroxy-6-methyl-2-oxopyridin-1(2H)-yl)ethane-1-sulfonamide **1i**

The mixture of 4-hydroxy-6-methyl-2-pyrone 5 (0.38 g, 3 mmol), sulfonamide hydrochloride 6 (0.48 g, 3 mmol), and NaOH (0.12 g, 3 mmol) in water (10 mL) was refluxed for 5 h. Then, the precipitate formed was collected by filtration, washed with water (3 × 10 mL), and dried to afford pure compound **1i**.

2-(4-hydroxy-6-methyl-2-oxopyridin-1(2H)-yl)ethane-1-sulfonamide **1i**: Yield 68%, m.p. 243–245 °C. ^1^H NMR (300 MHz, DMSO-*d*_6_) δ 10.40 (br. s, 1H, OH); 7.88 (br. s, 2H, NH_2_); 5.89 (s, 1H, CH); 5.65 (s, 1H, CH); 4.25 (t, *J* = 6.2 Hz, 2H, CH_2_); 3.30 (t, *J* = 6.2 Hz, 2H, CH_2_); 2.39 (s, 3H, CH_3_). ^13^C NMR (300 MHz, DMSO-d6) δ 166.42, 164.15, 147.67, 101.16, 96.26, 56.47, 52.57, 38.86. Anal. Calcd. for C_8_H_12_N_2_O_4_S (%)—C, 41.37; H, 5.21; N, 12.06. Found (%)—C, 41.32; H, 5.15; N, 12.13.

#### 3.1.4. Synthesis of 2-(((2,4-Dioxochroman-3-ylidene)methyl)amino)ethane-1-sulfonamide **1j**

The mixture of 4-hydroxycoumarin (0.49 g, 3 mmol), sulfonamide hydrochloride 6 (0.48 g, 3 mmol), and Et_3_N (0.3 g, 3 mmol) in triethyl orthoformate (7 mL) was refluxed for 6 h. Then, obtained solution was evaporated and residue was recrystallized from EtOH (5 mL). The precipitate formed was collected by filtration, washed with EtOH (3 × 5 mL), and dried to afford pure compound **1j**.

2-(((2,4-dioxochroman-3-ylidene)methyl)amino)ethane-1-sulfonamide **1j**: Yield 47%, m.p. 197–199 °C. Mixture of E- and Z-isomers. ^1^H NMR (300 MHz, DMSO-*d*_6_) δ 11.80-11.62 (m, 0.7H, NH); 10.49–10.31 (m, 0.3H, NH); 8.61 (d, *J* = 14.7 Hz, 0.3H, CH); 8.49 (d, *J* = 14.7 Hz, 0.7H, CH); 8.00–7.91 (m, 1H, CH); 7.62–7.53 (m, 1H, CH); 7.29–7.18 (m, 2H, 2CH); 6.91 (br. s, 2H, NH_2_); 4.10–3.98 (m, 2H, CH_2_); 3.45–3.34 (m, 2H, CH_2_). ^13^C NMR (300 MHz, DMSO-d6) δ 179.72, 177.52, 163.58, 163.43, 163.14, 161.94, 154.65, 154.59, 134.87, 134.80, 126.21, 125.74, 124.50, 124.40, 120.70, 117.46, 117.35, 96.33, 56.46, 54.21, 46.21, 46.16. Anal. Calcd. for C_12_H_12_N_2_O_5_S (%)—C, 48.64; H, 4.08; N, 9.45. Found (%)—C, 48.59; H, 4.03; N, 9.48.

#### 3.1.5. Synthesis of 2-Methyl-4-oxo-3-(4-sulfamoylphenethyl)-3,4,5,6-tetrahydro-2H-2,6-methanobenzo[g] [1,3]oxazocine-5-carboxamide **1k**

The mixture of chromene-3-carboxamide 8 (0.57 g, 3 mmol) and sulfonamide 9 (0.6 g, 3 mmol) in acetone (5 mL) and MeOH (5 mL) was refluxed for 16 h. Then, obtained solution was evaporated and residue was recrystallized from MeOH (4 mL). The precipitate formed was collected by filtration, washed with MeOH (3 × 5 mL), and dried to afford pure compound **1k**.

2-methyl-4-oxo-3-(4-sulfamoylphenethyl)-3,4,5,6-tetrahydro-2H-2,6-methanobenzo[g][1,3]oxazocine-5-carboxamide **1k**: Yield 56%, m.p. 225–227 °C. ^1^H NMR (300 MHz, DMSO-*d*_6_) δ1H NMR (500 MHz, DMSO-d6) δ 7.72 (d, J = 7.8 Hz, 2H, H-19, H-20); 7.62 (s, 1H, NH); 7.41 (d, *J* = 6.56 Hz, 2H, H-25, H-26); 7.33 (d, *J* = 6.08 Hz, 1H, H-28); 7.27 (s, 3H, NH_2_, NH); 7.14 (td, *J_1_* = 6.0 Hz, *J_2_* = 1.6 Hz, 1H, H-30); 6.94 (t, *J* = 7.4 Hz, 1H, H-29); 6.76 (d, *J* = 8.1 Hz, 1H, H-27); 4.33 (t, *J* = 5.1 Hz, 1H, H-4); 3.60–3.53 (m, 1H, 12a); 3.48–3.40 (m, 1H, 12b); 2.91–2.83 (m, 1H, H-23b); 2.74–2.66 (m, 1H, H-23a); 2.05 (dd, *J_1_* = 10.84 Hz, *J_2_* = 1.12 Hz, 1H, H-8b’’); 1.73 (s, 3H, H-22); 1.04 (t, 1H, H-8a’’). ^13^C NMR (300 MHz, DMSO-d6) δ 3: 170.40; 1: 167.80; 8: 151.45; 16: 143.90; 19: 142.61; 12: 129.59; 9: 129.09; 17: 129.55; 18: 126.22; 10: 124.94; 11: 121.84; 13: 117.38; 4: 86.22; 7: 57.38; 14: 56.48; 6: 43.09; 4: 34.63; 4: 34.73; 15: 31.94; 2: 30.94; 5.18.99. Anal. Calcd. for C_21_H_23_N_3_O_5_S (%)—C, 58.73; H, 5.40; N, 9.78. Found (%)—C, 58.82; H, 5.37; N, 9.80.

### 3.2. Molecular Docking Studies

Molecular modeling studies were performed using AutoDock 4.2 software [70]. Protein Data Bank was also used in order to obtain the crystal structures of hCA I (PDB code: 3W6H) and hCA II (PDB code: 3HS4) cytosolic isoforms, hCA IX (PDB code: 3IAI) and hCA XII (PDB code: 1JD0) transmembrane tumor-associated isoforms, and *E. coli* β-carbonic anhydrase (PDB code: 1IP6) and γ-carbonic anhydrase (PDB code: 1QRL) [71]. All the procedures were carried out as in our previous work [53]. 

### 3.3. CA Inhibition Assay

An Applied PhotoPhysics stopped-flow instrument was used for assaying the CA-catalyzed CO_2_ hydration activity. Phenol red (at a concentration of 0.2 mM) was used as an indicator, working at the absorbance maximum of 557 nm, with 20 mM Hepes (pH 7.4) as a buffer for α-class and 20 mM TRIS (pH 8.3) as a buffer for β- and γ-class, and 20 mM Na_2_SO_4_ (for maintaining constant ionic strength), following the initial rates of the CA-catalyzed CO_2_ hydration reaction for a period of 10–100 s. The CO_2_ concentrations ranged from 1.7 to 17 mM for the determination of the kinetic parameters and inhibition constants. The uncatalyzed CO_2_ hydration was not subtracted from these curves and accounts for the remaining observed activity even at a high concentration of inhibitor, being in the range of 16–25%. However, the background activity from the uncatalyzed reaction was always subtracted when IC_50_ values were obtained by using the data analysis software for the stopped-flow instrument. Enzyme concentrations ranged between 5 and 10 nM. For each inhibitor, at least six traces of the initial 5–10% of the reaction were used for determining the initial velocity. The uncatalyzed rates were determined in the same manner and subtracted from the total observed rates. Stock solutions of the inhibitor (0.1 mM) were prepared in distilled–deionized water, and dilutions up to 0.01 nM were carried out thereafter with the assay buffer. Inhibitor and enzyme solutions were preincubated together for 15 min at room temperature prior to the assay to allow for the formation of the E–I complex. The inhibition constants were obtained by nonlinear least-squares methods using PRISM 3 and the Cheng–Prusoff equation, as reported earlier, and represent the mean from at least three different determinations. All CA isoforms were recombinant proteins obtained in house, as reported earlier [3,72,73,74].

### 3.4. Drug-Likness

The study was performed as described in our previous paper [52].

## 4. Conclusions

In conclusion, we synthetized and investigated a novel series of pyrazolo[4,3-c]pyridine sulfonamides for their effective inhibition against the most relevant human carbonic anhydrase isoforms, such as the ubiquitous hCA I and hCA II isoforms and the tumor-associated isoforms hCA IX and XII, which are implicated in many diseases such as glaucoma, retinitis pigmentosa, epilepsy, and tumors. Furthermore, the inhibitory activity against 3β-CAs and 3γ-CAs from different bacterial strains were evaluated. Five out of 11 compounds (**1b**, **1f**, **1g**, **1h** and **1k**) were more potent than AAZ, while compounds **1f** and **1k** showed better activity than the reference drug against the hCA II isoform. It should be mentioned that these two compounds were the most selective, with selectivity indexes of 9 and 15.8 towards the hCA I isoform and 71.9 and 6 towards the hCA XII isoform, respectively. As far as the inhibition of the 3β- and 3γ-CAs from different bacterial strains is concerned, in general, the compounds showed good activity. Thus, nine out of eleven were more potent than AAZ against *E.coli* γ, eight against *Bps*CA β, and seven against *Pgi*CA γ. Finally, compound **1b** was selective against all 3β-CA isoforms from *E.coli*, *BpsCA*, and *VhCA* and all 3γ-CA isoforms from *E.coli*, *BpsCA,* and *PgiCA,* with selectivity indexes (SI) of 59.8, 3.8, 14.6, 8.88, and 1.5, respectively. Furthermore, computational procedures were used to investigate the binding mode of this class of compounds, and the results were in agreement with the experimental data.

## Figures and Tables

**Table 1:** Structure of synthesized compounds **1a–1k**.

Compound	Structure	Compound	Structure
**1a**	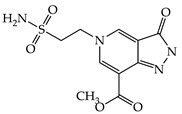	**1g**	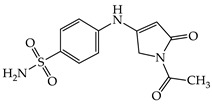
**1b**	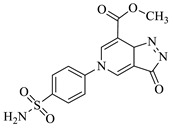	**1h**	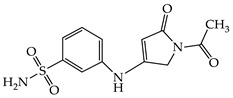
**1c**	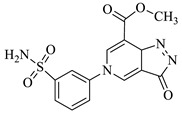	**1i**	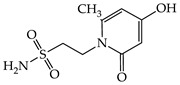
**1d**	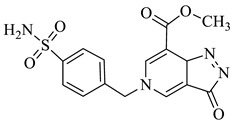	**1j**	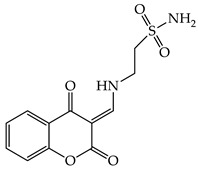
**1e**	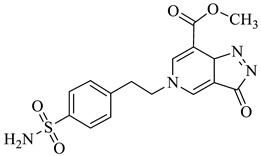	**1k**	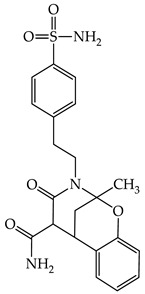
**1f**	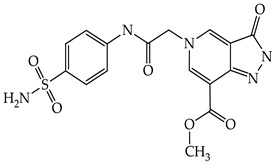		

**Table 2:** Inhibition data of human CA isoforms I, II, IX, and XII with labeled compounds and AAZ by a stopped-flow CO_2_ hydrase assay.

K_I_ (nM) *				
Cmp	hCA I	hCAII	hCA IX	hCA XII
**1a**	8010	7329	97.9	282.3
**1b**	156.8	51.4	319.1	358.2
**1c**	1443	247.4	589.5	143.2
**1d**	847.7	779.3	644.7	262.4
**1e**	864.2	658.3	848.8	397.4
**1f**	58.8	6.6	907.5	474.8
**1g**	66.8	41.7	294.2	508.5
**1h**	135.8	61.7	94.3	713.6
**1i**	5439	6791	79.6	104.8
**1j**	3865	5712	97.8	285.1
**1k**	88.3	5.6	421.4	34.5
**AAZ**	250.0	12.1	25.8	5.7

* Mean from 3 different assays, by a stopped flow technique (errors were in the range of ±5–10% of the reported values).

**Table 3:** Inhibition data against different β- and γ-CA isoforms from *E. coli*, *BpsCA*, *PgiCA, and VhCA* for the labeled compounds and AAZ by a stopped-flow CO_2_ hydrase assay.

K_I_ (nM) *						
Cmp	*E.coli* β	*E.coli* γ	*Bps*CAβ	*Bps*CAγ	*Pgi*CAγ	*Vh*CAβ
**1a**	861.9	61.8	654.3	912.8	783.0	2334
**1b**	3457	57.8	229.1	513.2	91.0	844.2
**1c**	3836	79.1	785.4	613.5	637.1	913.3
**1d**	5027	189.7	644.4	805.1	848.9	670.7
**1e**	3136	58.1	682.9	1341	96.1	840.0
**1f**	3650	66.8	236.3	2179	667.8	466.6
**1g**	453.8	204.7	664.3	97.1	83.1	1449
**1h**	711.9	524.3	2961	833.2	90.0	2617
**1i**	3048	92.7	96.4	191.5	95.6	2241
**1j**	94.9	67.1	788.8	625.4	84.3	642.3
**1k**	3864	63.5	212.5	952.3	201.6	355.8
**AAZ**	227	248	745	149	324	451

* Mean from 3 different assays by a stopped-flow technique

**Table 4:** Molecular docking free binding energies (kcal/mol) and interactions of tested compounds on hCA I, II, IX, and XII isoforms.

No	*h*CA Isoform	Estimated Free Binding Energy (Kcal/mol)	Chelating the Zn (II) Ion	Residues Involved in H-Bond Interactions	Residues Involved in Hydrophobic Interactions
**1c**	hCA I	−4.70	No	-	-
	hCA II	−5.03	No	-	Ile91, Phe131
	hCA IX	−6.06	Yes	Thr199	Val121, Leu198
	hCA XII	−5.92	Yes	-	Leu198
**1g**	hCA I	−10.42	Yes	Trp5, Thr199, His200	Leu198, His200
	hCA II	−6.89	Yes	Thr199	Val121, Leu198
	hCA IX	−7.65	Yes	Thr199, Thr200	Leu198
	hCA XII	6.11	Yes	Thr200	Trp5, Leu198
**1f**	hCA I	−11.37	Yes	Trp5, Ser136, Thr199	Ala121, Leu198
	hCA II	−10.12	Yes	Gln92, Thr199	Val121, Leu198, Thr200
	hCA IX	−4.29	Yes	-	Val121, Leu198
	hCA XII	−5.50	Yes	Gln92	Val121, Leu198
**1k**	hCA I	−9.25	Yes	Thr199, His200	Leu198, His200
	hCA II	−10.53	Yes	Gln92, Thr199 (2)	Val121, Phe131, Val135, Leu198
	hCA IX	−6.17	Yes	-	Val121, Leu198
	hCA XII	−6.79	Yes	Thr199	Val121, Leu198, Trp209
**AAZ**	hCA I	−8.28	Yes	Gln92	Leu198, Thr199, His200, Pro201, Trp209
	hCA II	−8.87	Yes	Thr199, Thr200	Val121, Phe131, Leu198, Trp209
	hCA IX	−9.02	Yes	Thr199, Thr200	Val121, Val143, Val131, Leu198, Trp209
	hCA XII	−9.14	Yes	Thr199, Thr200	Val121, Val143, Leu198, Trp209

**Table 5:** Molecular docking free binding energies (kcal/mol) and interactions of tested compounds on β- and γ-CA classes.

No	*h*CA Isoform	Estimated Free Binding Energy (Kcal/mol)	Chelating The Zn (II) Ion	Residues Involved in H-Bond Interactions	Residues Involved in Hydrophobic Interactions
**1a**	*E. coli* β	−3.15	No	-	-
	γ	−5.18	No	-	Leu80, Ala82
**1b**	*E. coli* β	−1.07	No	-	-
	γ	−10.86	Yes	Gln120, H_2_O	Val79
**1c**	*E. coli* β	−2.40	No	-	-
	γ	−7.52	Yes	Ser57	Val78
**1d**	*E. coli* β	−1.66	No	-	-
	γ	-	No	-	-
**1e**	*E. coli* β	−2.71	No	-	-
	γ	−10.57	Yes	H_2_O	Val78, Val79
**1f**	*E. coli* β	-	No	-	-
	γ	−9.16	Yes	Ser57, Arg59	Val79, Leu83
**1g**	*E. coli* β	−3.16	No	-	Ala106
	γ	−2.55	No	-	Val78
**1h**	*E. coli* β	−3.02	No	-	Ile126
	γ	−2.61	No	-	Val78
**1i**	*E. coli* β	−1.28	No	-	-
	γ	−7.43	Yes	Glu62	Val79
**1j**	*E. coli* β	−8.61	Yes	Gly103	Ala106
	γ	−10.35	Yes	Arg59, H_2_O	Val79
**1k**	*E. coli* β	−2.58	No	-	-
	γ	−10.59	Yes	Arg59, H_2_O	Val78
**AAZ**	*E. coli* β	−3.46	No	-	Ala106, Val198
	γ	−4.27	No	Glu140	-

**Table 6:** Drug-likeness predictions of tested compounds.

Cmp	MW	Number of HBA ^a^	Number of HBD ^b^	Log *P*_o/w_ (iLOGP) ^c^	Log S ^d^	TPSA ^e^	Lipinski Violations	Bioavailability Score	Drug-Likeness Model Score
**1a**	300.29	7	2	−0.01	Very soluble	145.52	0	0.55	−0.43
**1b**	348.33	7	2	1.36	Soluble	145.52	0	0.55	−0.47
**1c**	348.33	7	2	0.92	Soluble	145.52	0	0.55	−0.94
**1d**	362.36	7	2	1.45	Moderately soluble	145.52	0	0.55	−0.08
**1e**	376.39	7	2	1.74	Moderately soluble	145.52	0	0.55	−0.04
**1f**	405.39	8	3	0.57	Moderately soluble	174.62	1 *	0.55	0.83
**1g**	295.31	5	2	1.29	Very soluble	117.95	0	0.55	0.90
**1h**	294.31	6	1	−2.99	Very Soluble	105.92	0	0.55	−0.13
**1i**	232.26	5	2	0.16	Very soluble	105.92	0	0.55	−0.08
**1j**	296.30	6	2	0.57	Soluble	123.94	0	0.55	0.01
**1k**	429.49	6	2	1.09	Moderately soluble	141.17	0	0.55	0.44

(^a^) Number of hydrogen-bond acceptors; (^b^) number of hydrogen-bond donors; (^c^) lipophilicity; (^d^) water solubility (SILICOS-IT (S = Soluble)); (^e^) topological polar surface area (Å^2^); * Lipinsky N or O > 10.

## Data Availability

Data is contained within the article and supplementary material.

## Supplementary Materials

The following are available online at https://www.mdpi.com/article/10.3390/ph15030316/s1, Spectra of ^1^H-NMR and ^13^C-NMR.
