# Supplementary material for: Pyrazolo[4,3-c]pyridine Sulfonamides as Carbonic Anhydrase Inhibitors: Synthesis, Biological and In Silico Studies
Source: Pharmaceuticals (Basel). 2022 Mar 7;15(3):316. doi: 10.3390/ph15030316 (PMC8955975; doi:10.3390/ph15030316)
Supplement: Supplementary file 1 [file pharmaceuticals-15-00316-s001.zip › pharmaceuticals-1601855-supplementary.pdf]

^{1}H-NMR and ^{13}C-NMR of compounds

Compound 1a

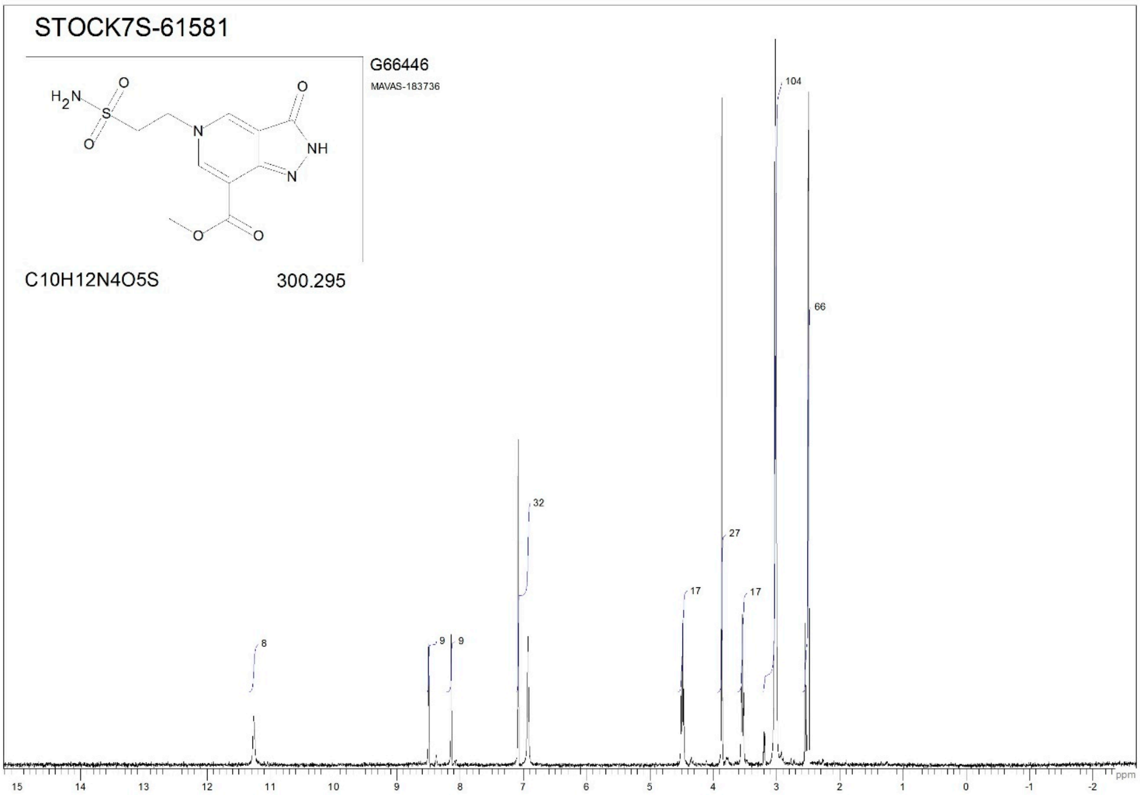

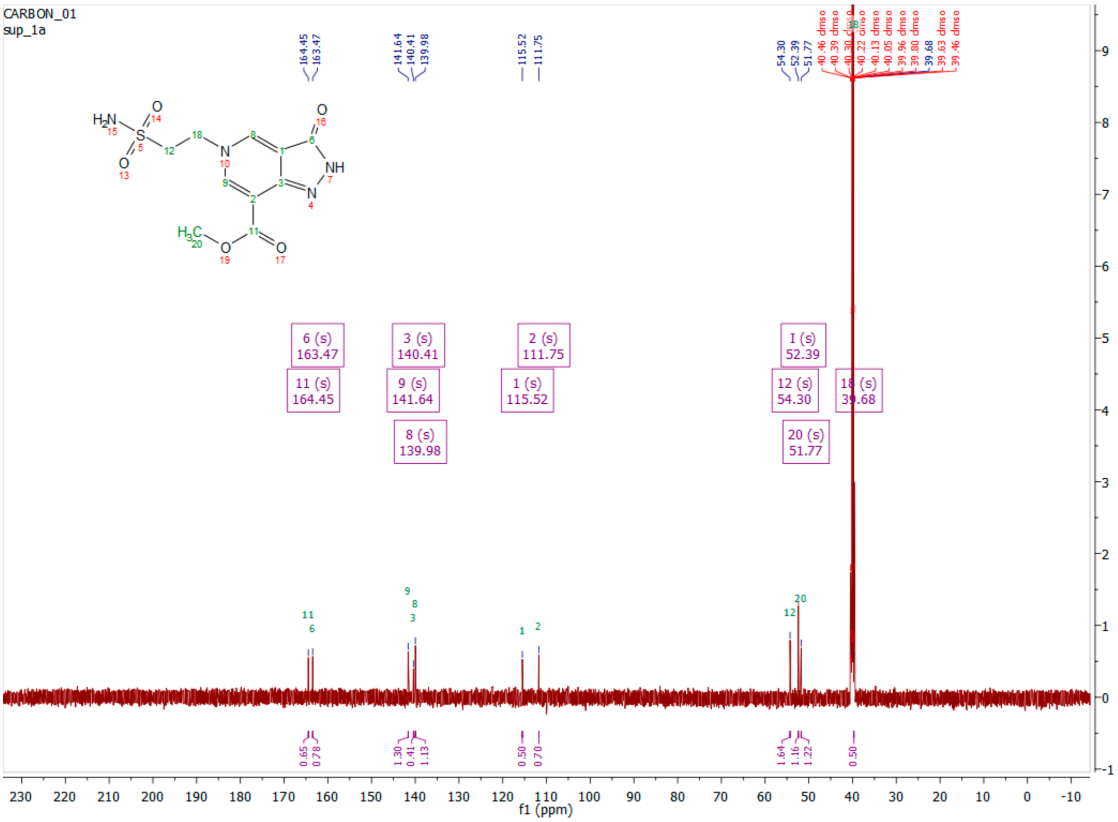

Compound 1b

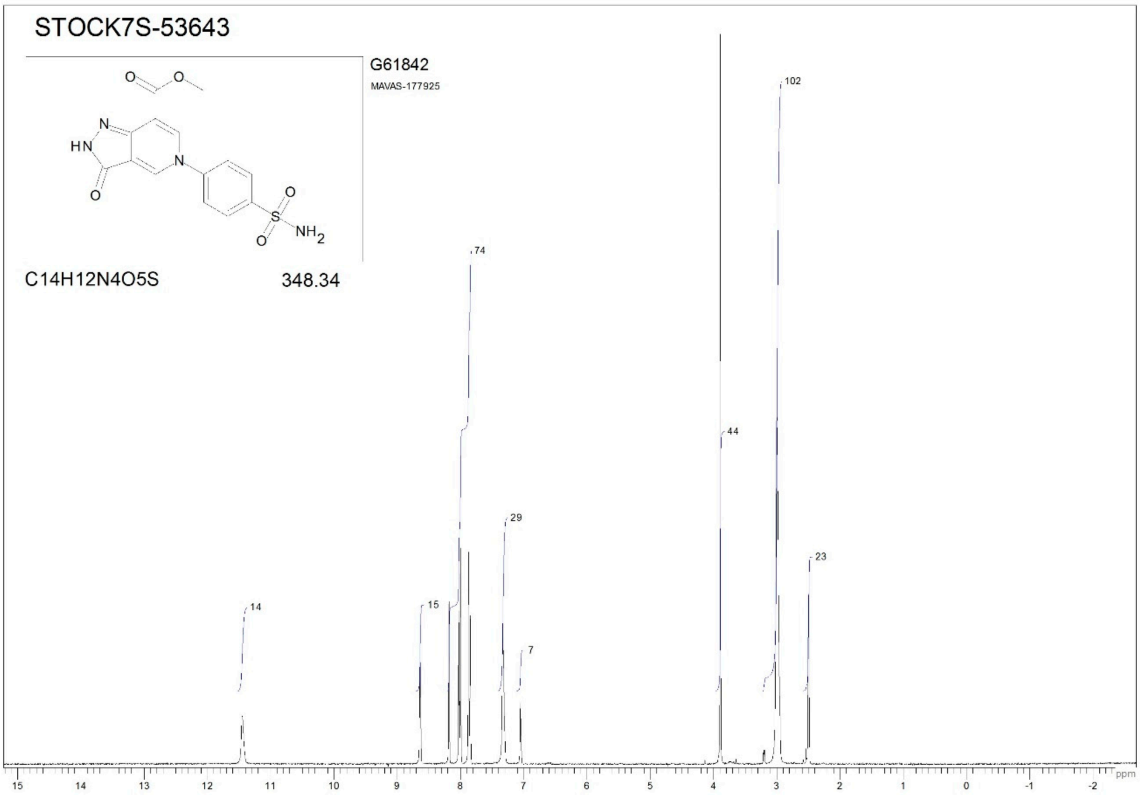

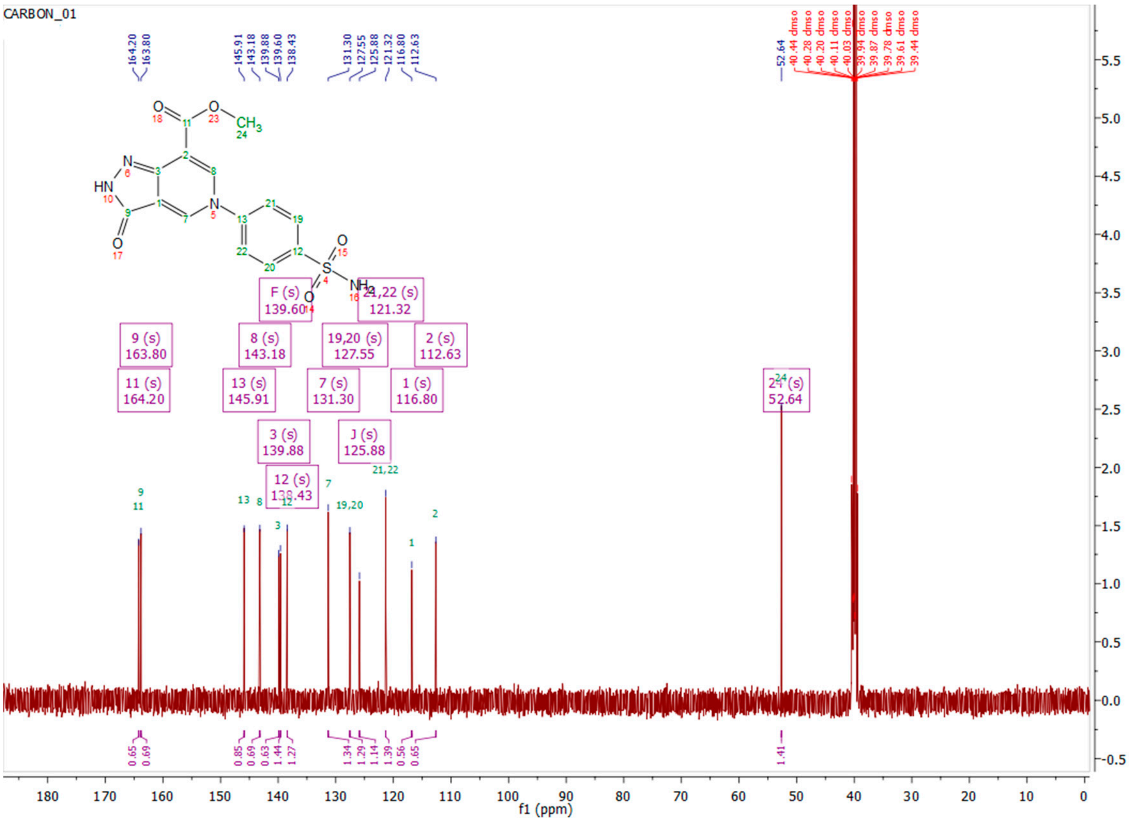

Compound 1c

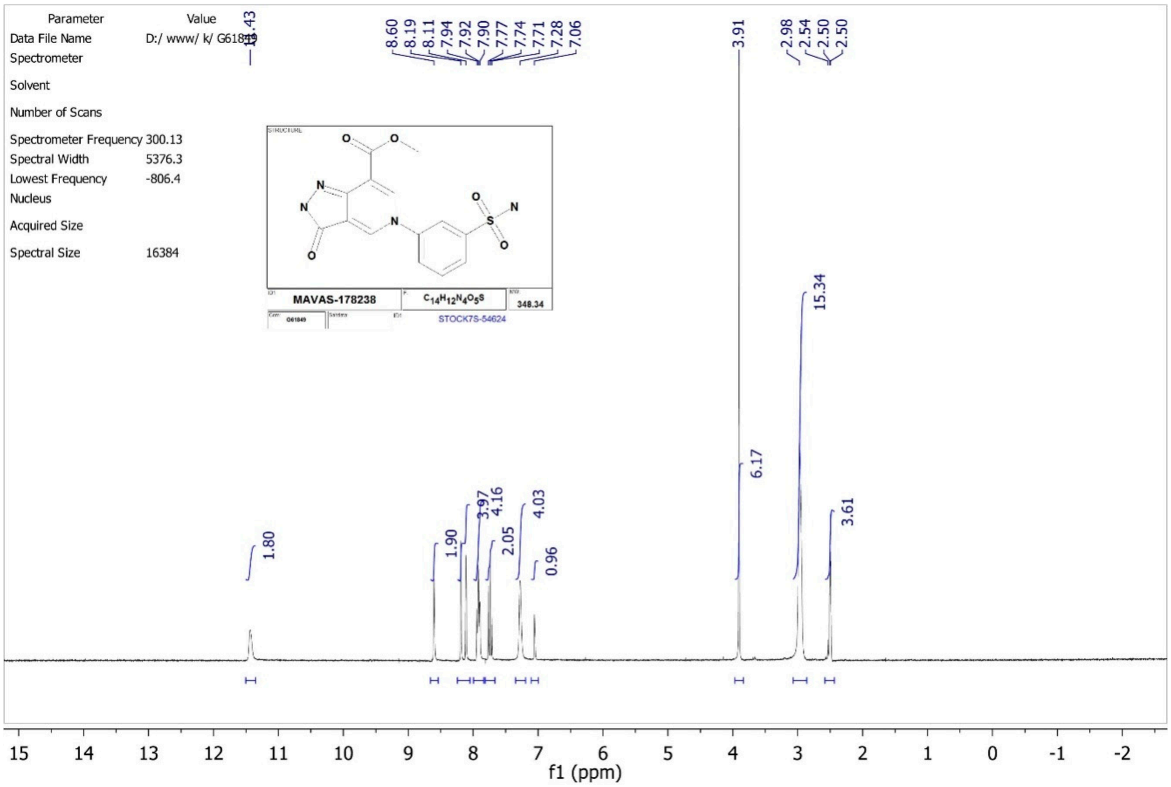
Parameter Value
Data File Name
Spectrometer
Solvent
Number of Scans
Spectrometer Frequency 300.13
Spectral Width 5376.3
Lowest Frequency -806.4
Nucleus
Acquired Size
Spectral Size 16384
11.43
8.60 8.19 8.11 7.94 7.92 7.90 7.77 7.74 7.71 7.28 7.06
3.91
2.98 2.54 2.50 2.50
MAVAS-178238
STOCK7S-54624
348.34
1.80
1.90
3.97
4.16
2.05
4.03
0.96
6.17
15.34
3.61
f1 (ppm)

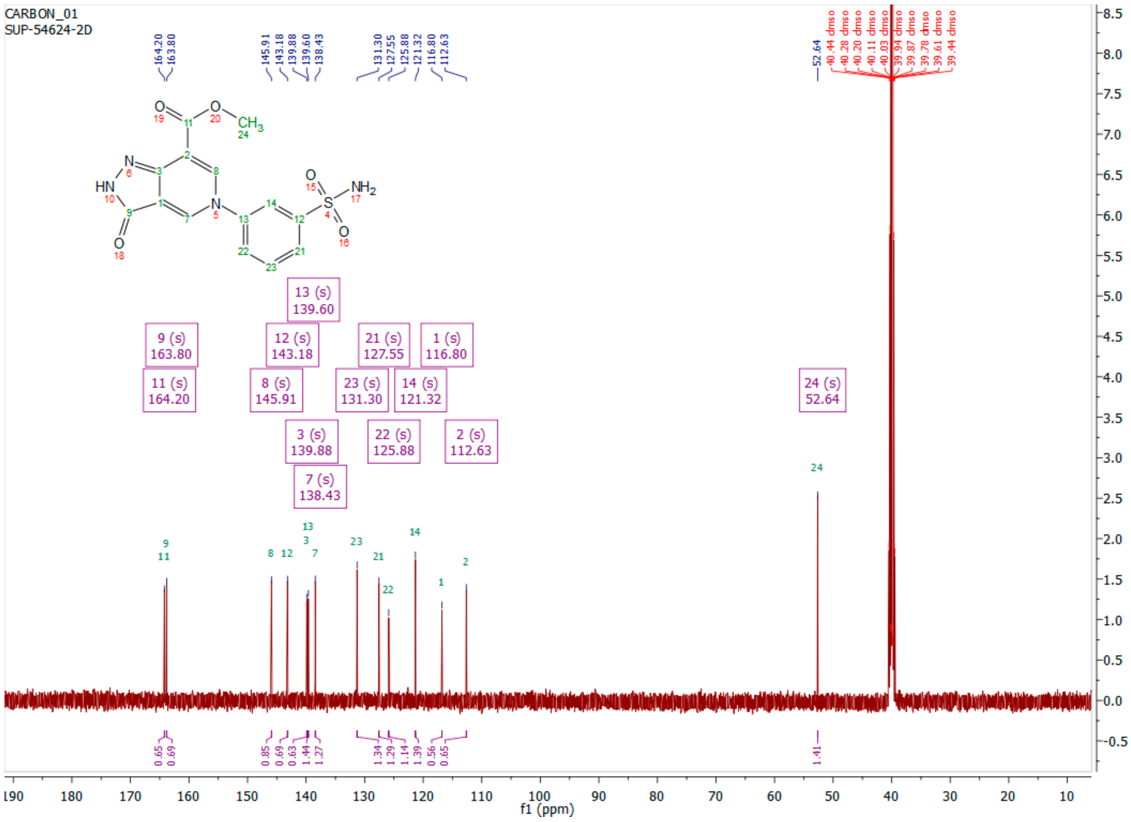
CARBON_01
SUP-54624-2D
164.20 163.80
145.91 143.18 139.88 139.60 138.43
131.30 127.55 125.88 121.32 116.80 112.63
52.64
13 (s) 139.60
9 (s) 163.80
12 (s) 143.18
21 (s) 127.55
1 (s) 116.80
11 (s) 164.20
8 (s) 145.91
23 (s) 131.30
14 (s) 121.32
24 (s) 52.64
3 (s) 139.88
22 (s) 125.88
2 (s) 112.63
7 (s) 138.43
f1 (ppm)

Compound 1d

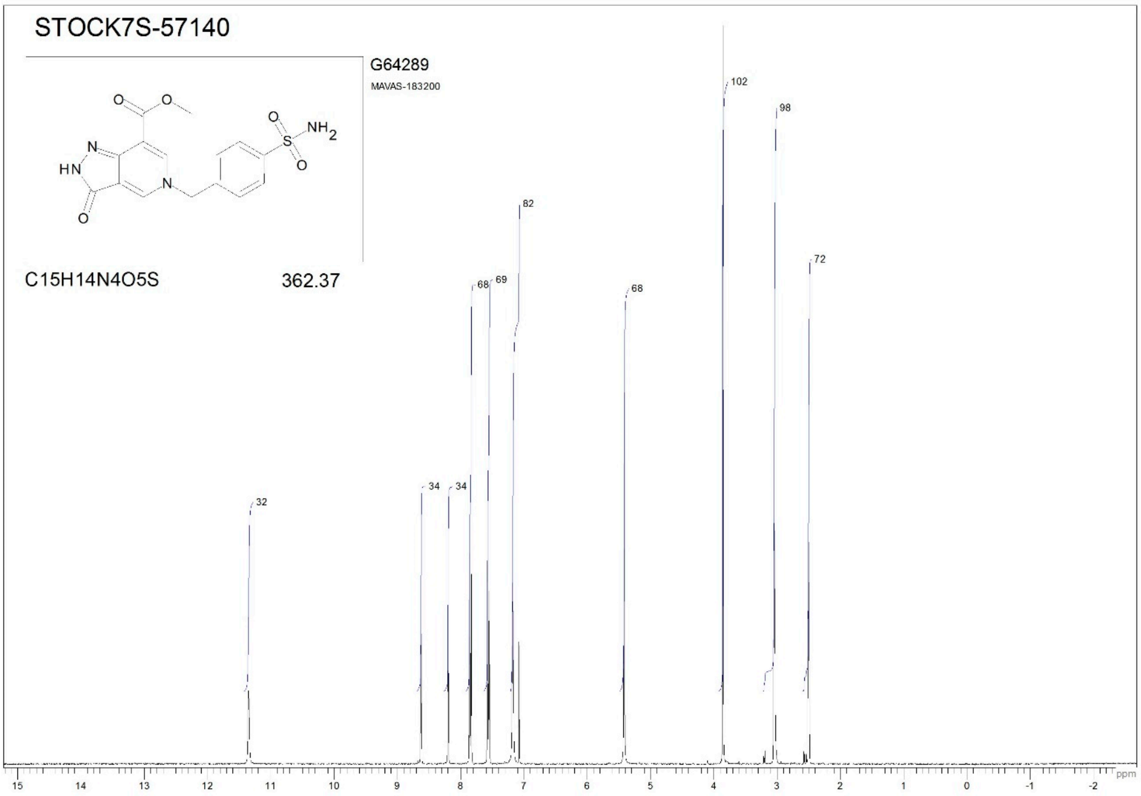
STOCK7S-57140
G64289
MAVAS-183200
C15H14N4O5S
362.37

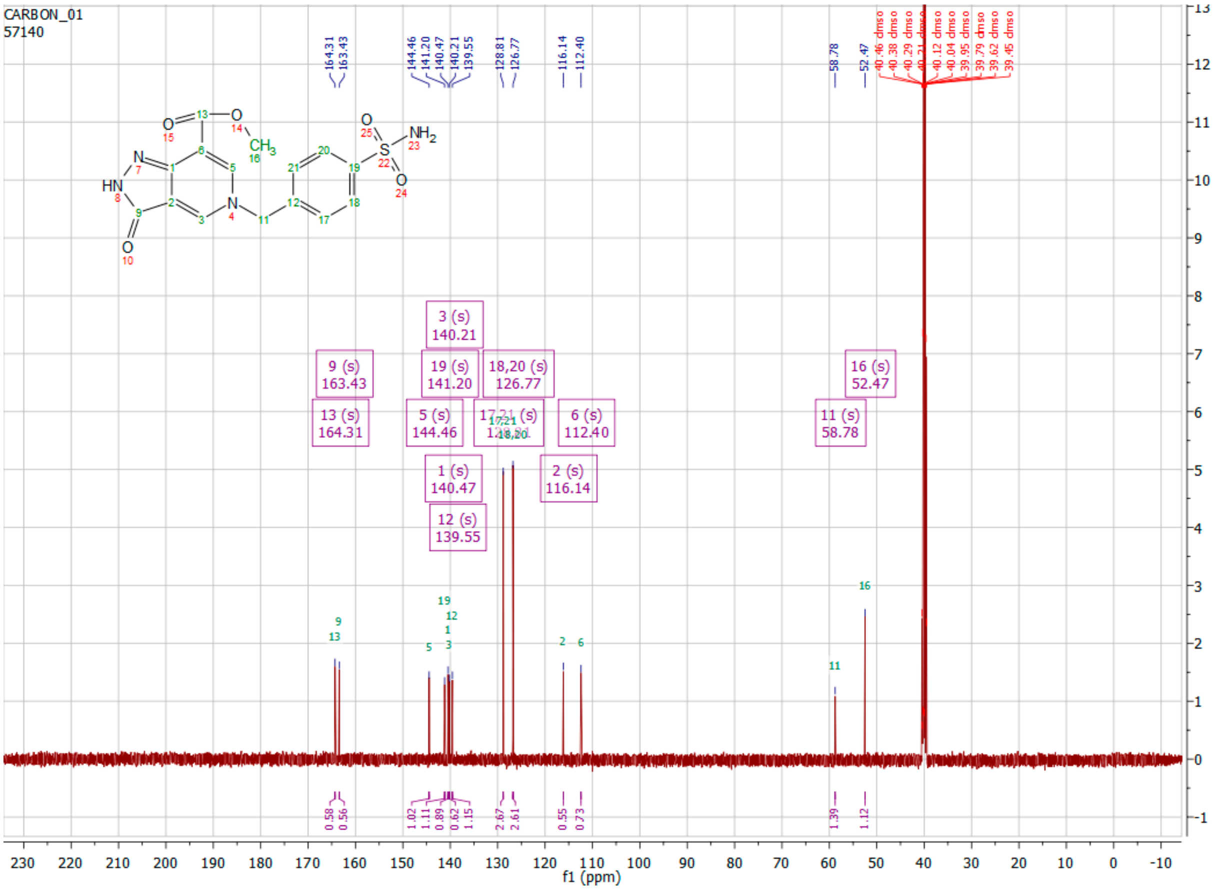
CARBON_01
57140
f1 (ppm)

Compound 1e

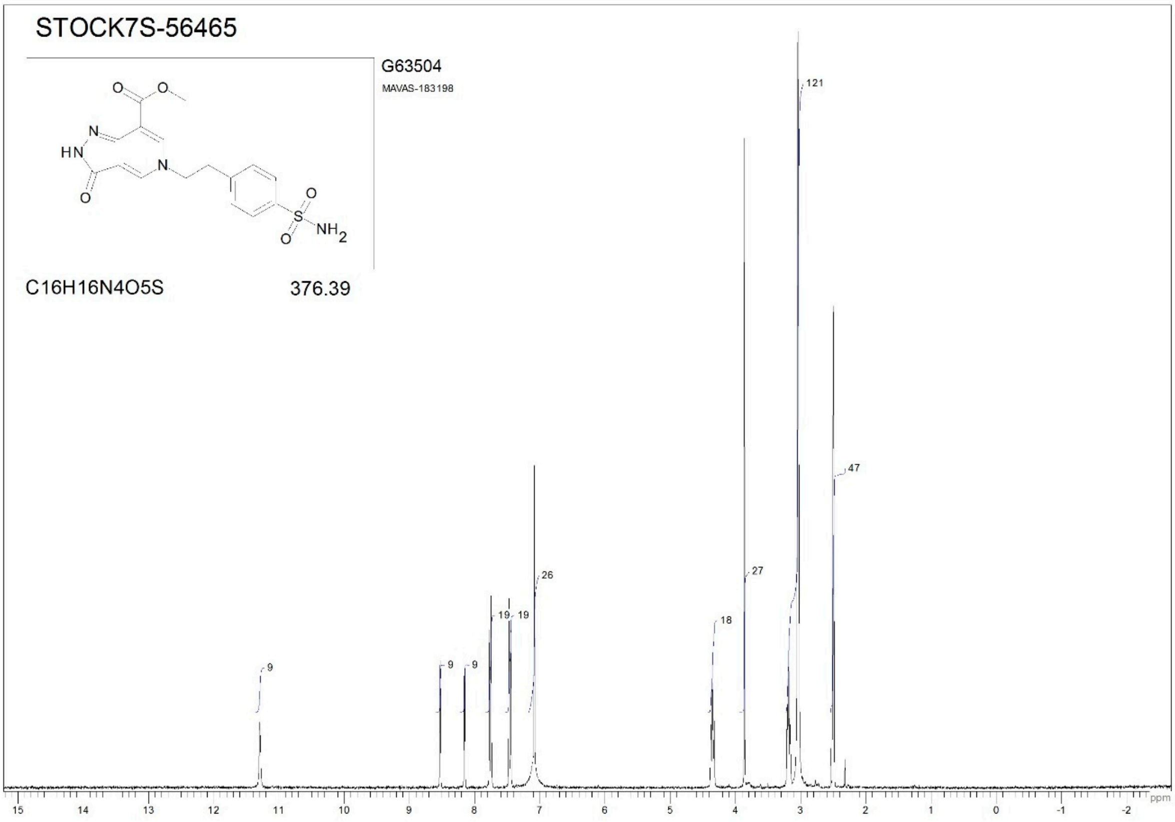
STOCK7S-56465
G63504
MAVAS-183198
C16H16N4O5S
376.39
ppm

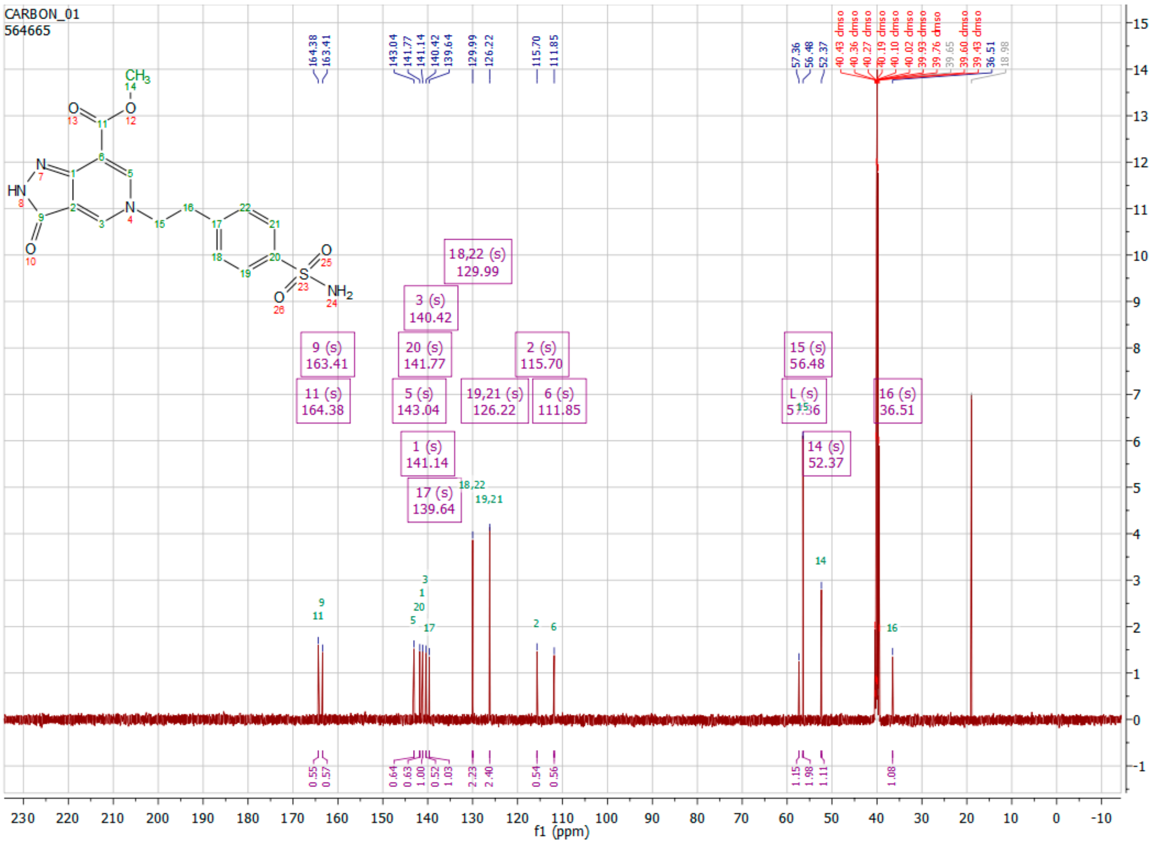
CARBON_01
564665
164.38
163.41
143.04
141.77
141.14
140.42
139.64
129.99
126.22
115.70
111.85
57.36
56.48
52.37
36.51
18,22 (s) 129.99
3 (s) 140.42
9 (s) 163.41
20 (s) 141.77
2 (s) 115.70
15 (s) 56.48
11 (s) 164.38
5 (s) 143.04
19,21 (s) 126.22
6 (s) 111.85
16 (s) 36.51
1 (s) 141.14
14 (s) 52.37
17 (s) 139.64
f1 (ppm)

Compound 1f

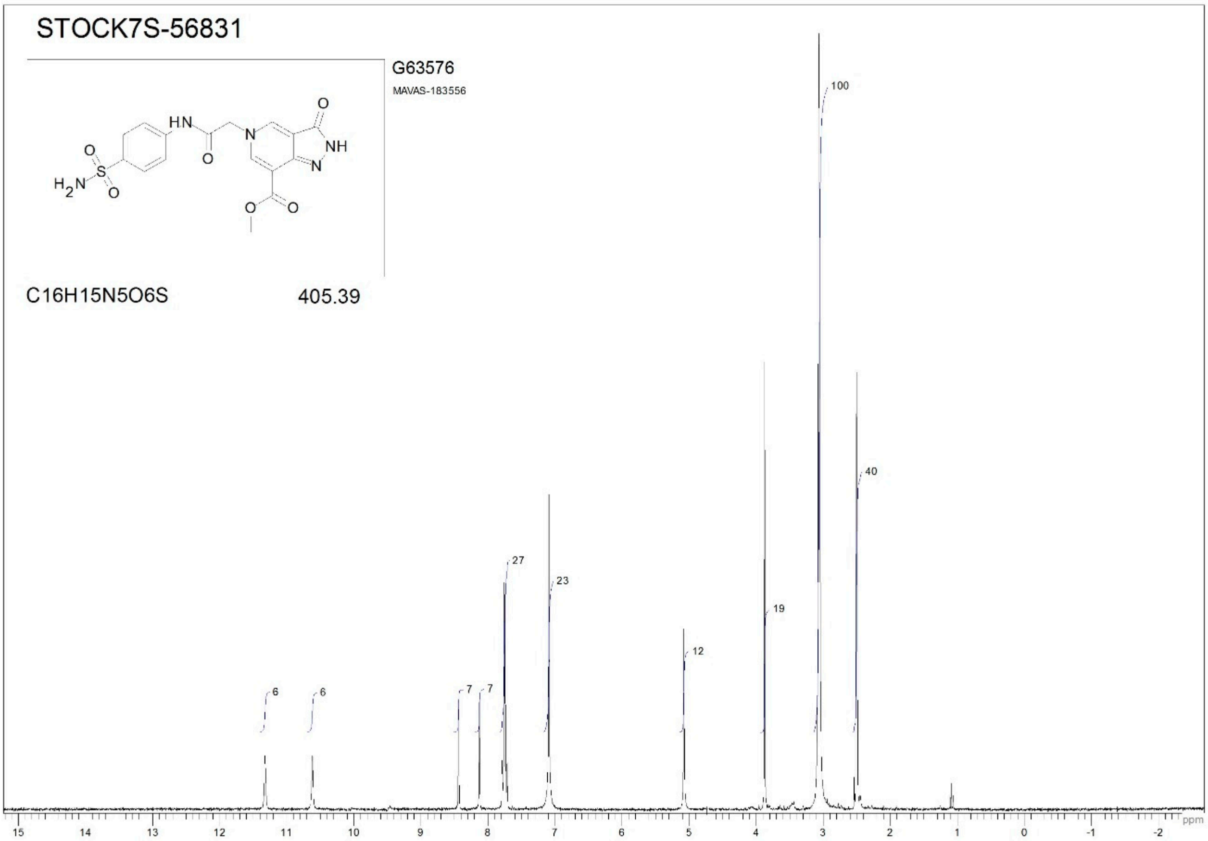
STOCK7S-56831
G63576
MAVAS-183556
C16H15N5O6S
405.39
100
40
27
23
19
12
6
6
7
7
ppm

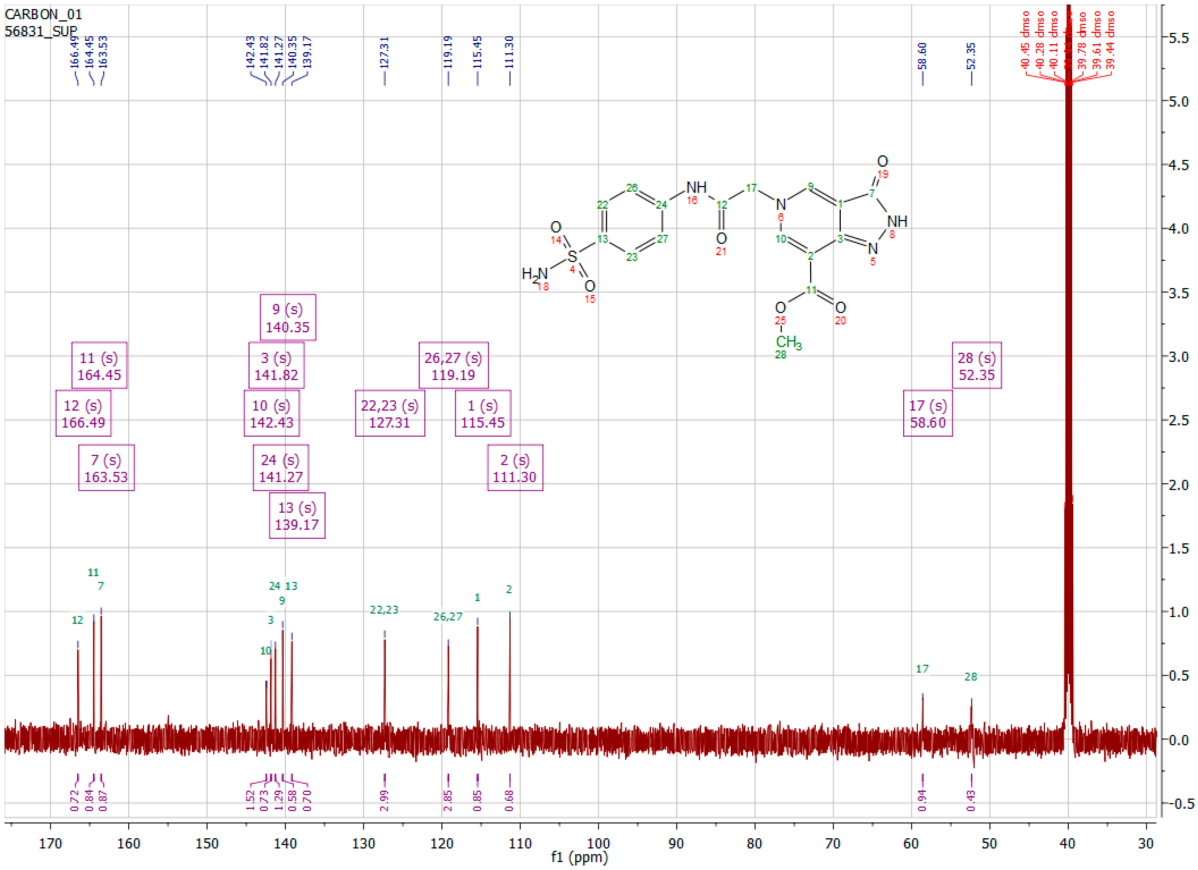
CARBON_01
56831_SUP
166.49
164.45
163.53
142.43
141.82
141.27
140.35
139.17
127.31
119.19
115.45
111.30
58.60
52.35
40.45 dmso
40.28 dmso
40.11 dmso
39.78 dmso
39.61 dmso
39.44 dmso
9 (s) 140.35
11 (s) 164.45
3 (s) 141.82
26,27 (s) 119.19
28 (s) 52.35
12 (s) 166.49
10 (s) 142.43
22,23 (s) 127.31
1 (s) 115.45
17 (s) 58.60
7 (s) 163.53
24 (s) 141.27
2 (s) 111.30
13 (s) 139.17
0.72
0.84
0.87
1.52
0.73
1.29
0.58
0.70
2.99
2.85
0.85
0.68
0.94
0.43
f1 (ppm)

Compound 1g

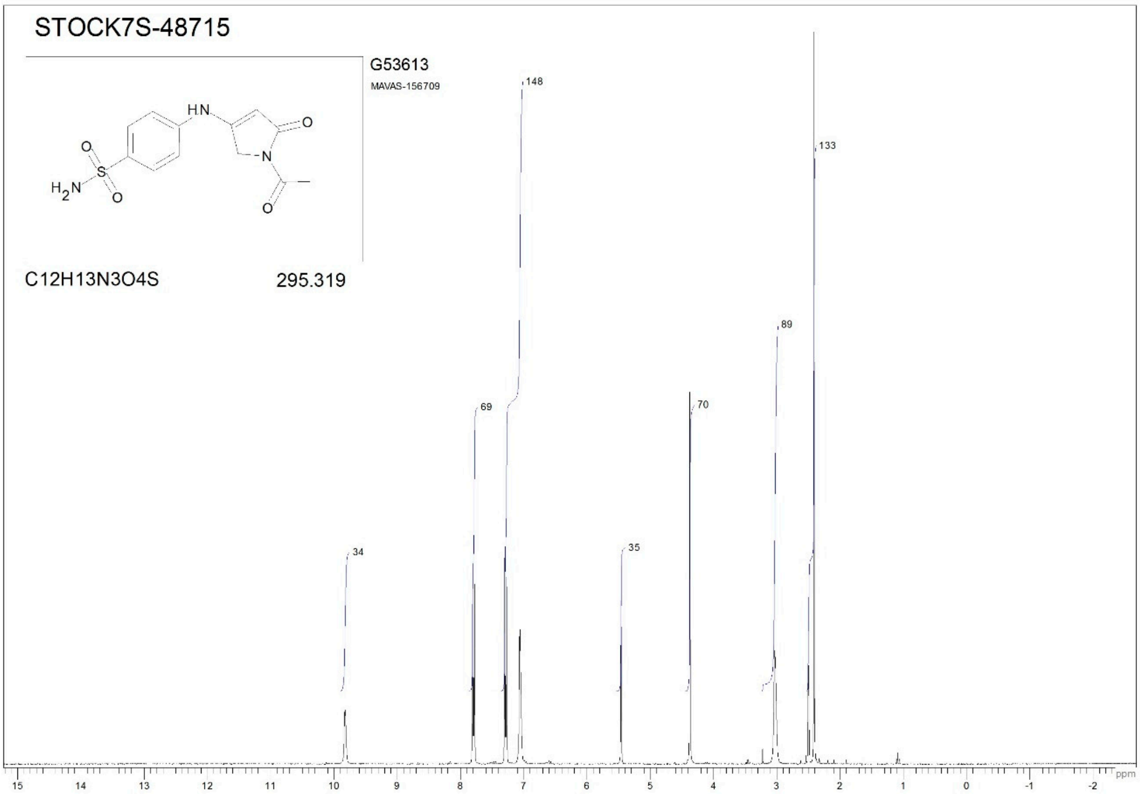

STOCK7S-48715
G53613
MAVAS-156709
C12H13N3O4S
295.319

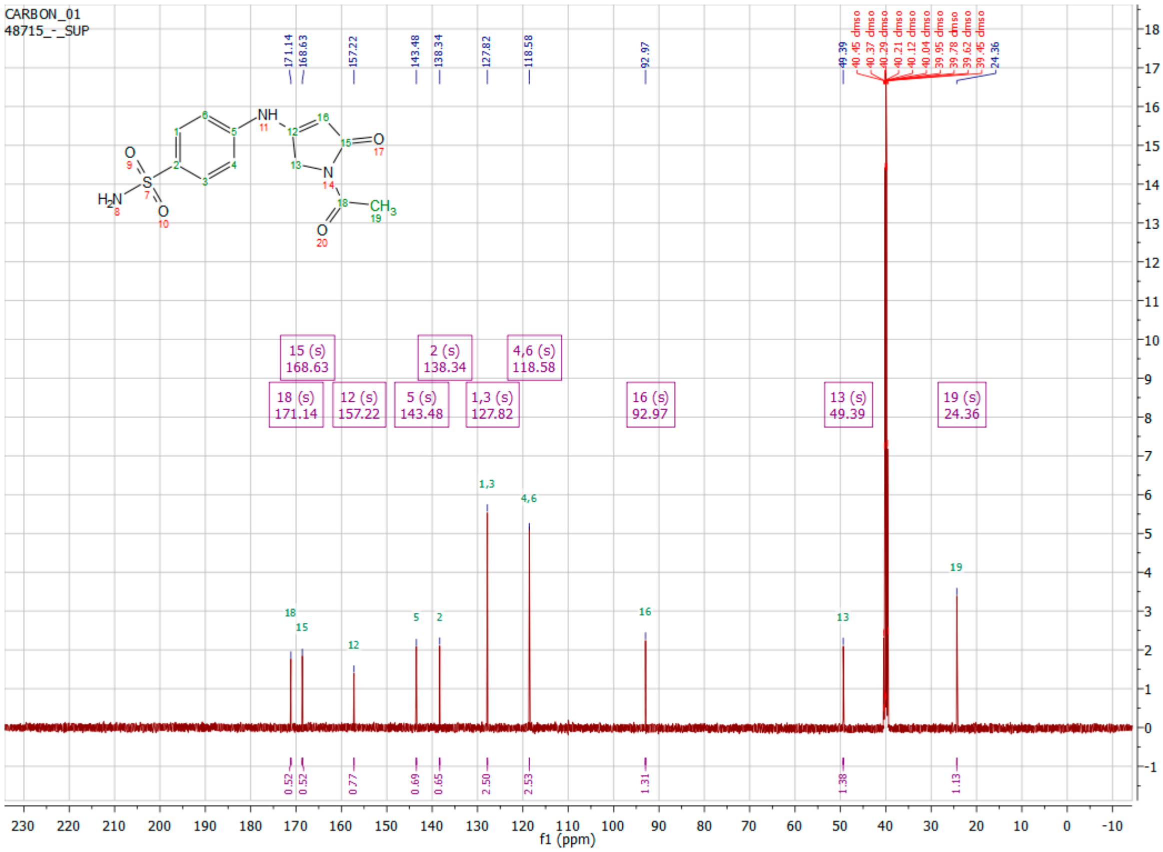

CARBON_01
48715_-_SUP
15 (s) 168.63
2 (s) 138.34
4,6 (s) 118.58
18 (s) 171.14
12 (s) 157.22
5 (s) 143.48
1,3 (s) 127.82
16 (s) 92.97
13 (s) 49.39
19 (s) 24.36
f1 (ppm)

Compound 1h

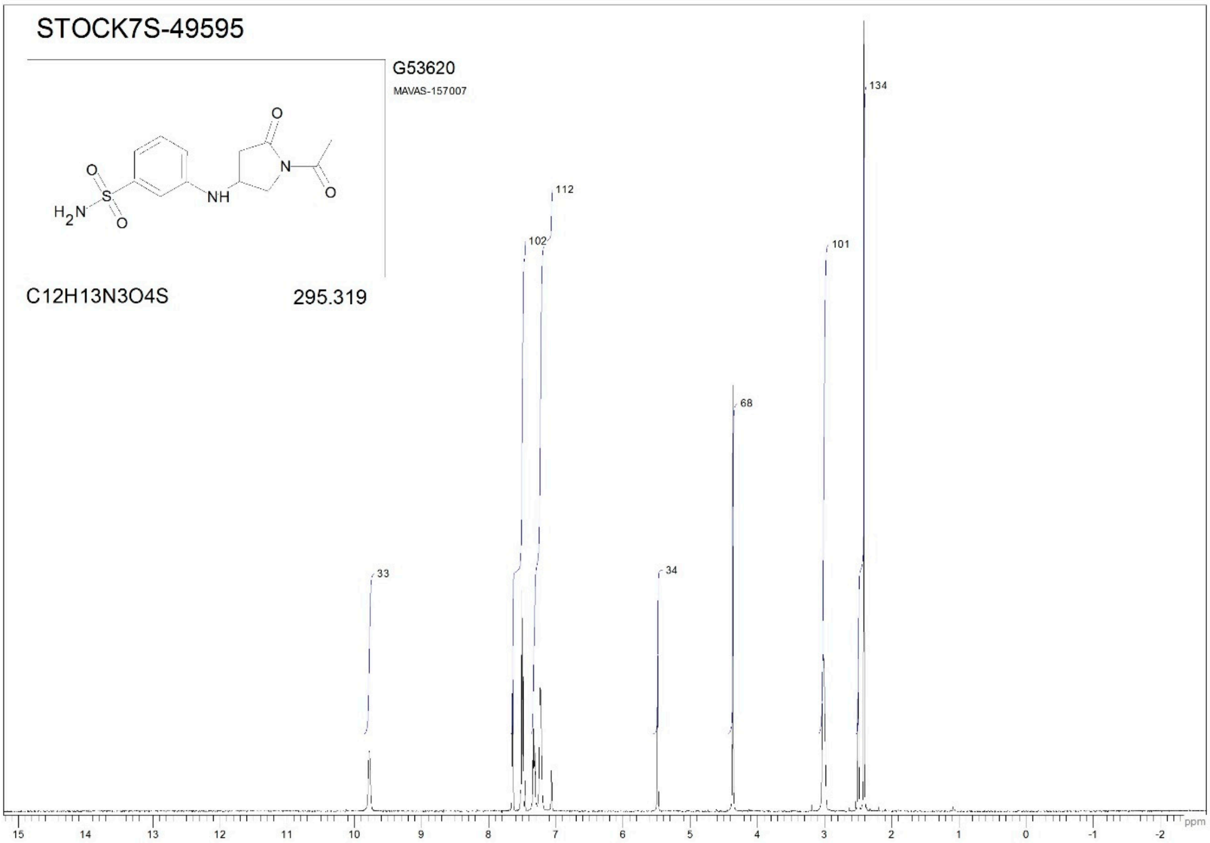
STOCK7S-49595
G53620
MAVAS-157007
C12H13N3O4S
295.319
ppm

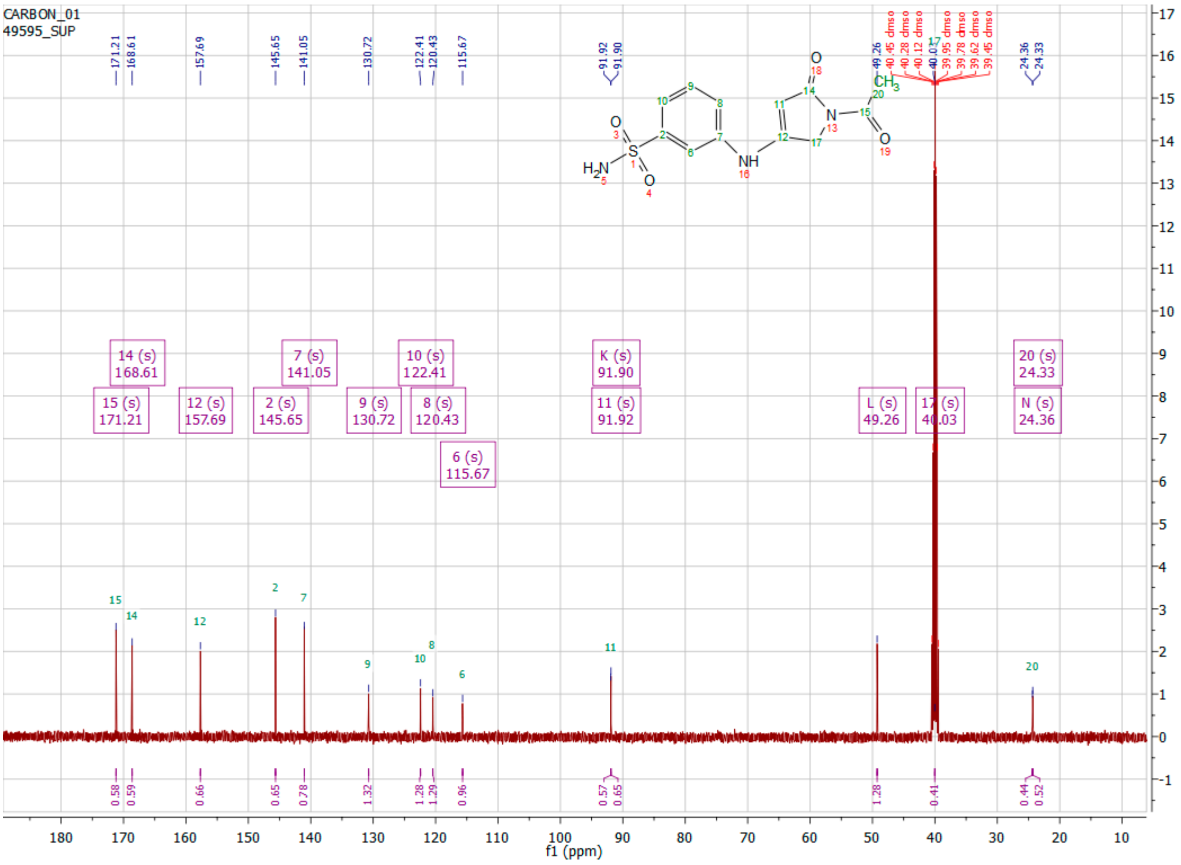
CARBON_01
49595_SUP
f1 (ppm)
14 (s) 168.61
15 (s) 171.21
12 (s) 157.69
7 (s) 141.05
2 (s) 145.65
9 (s) 130.72
10 (s) 122.41
8 (s) 120.43
6 (s) 115.67
K (s) 91.90
11 (s) 91.92
L (s) 49.26
17 (s) 40.03
20 (s) 24.33
N (s) 24.36

Compound 1i

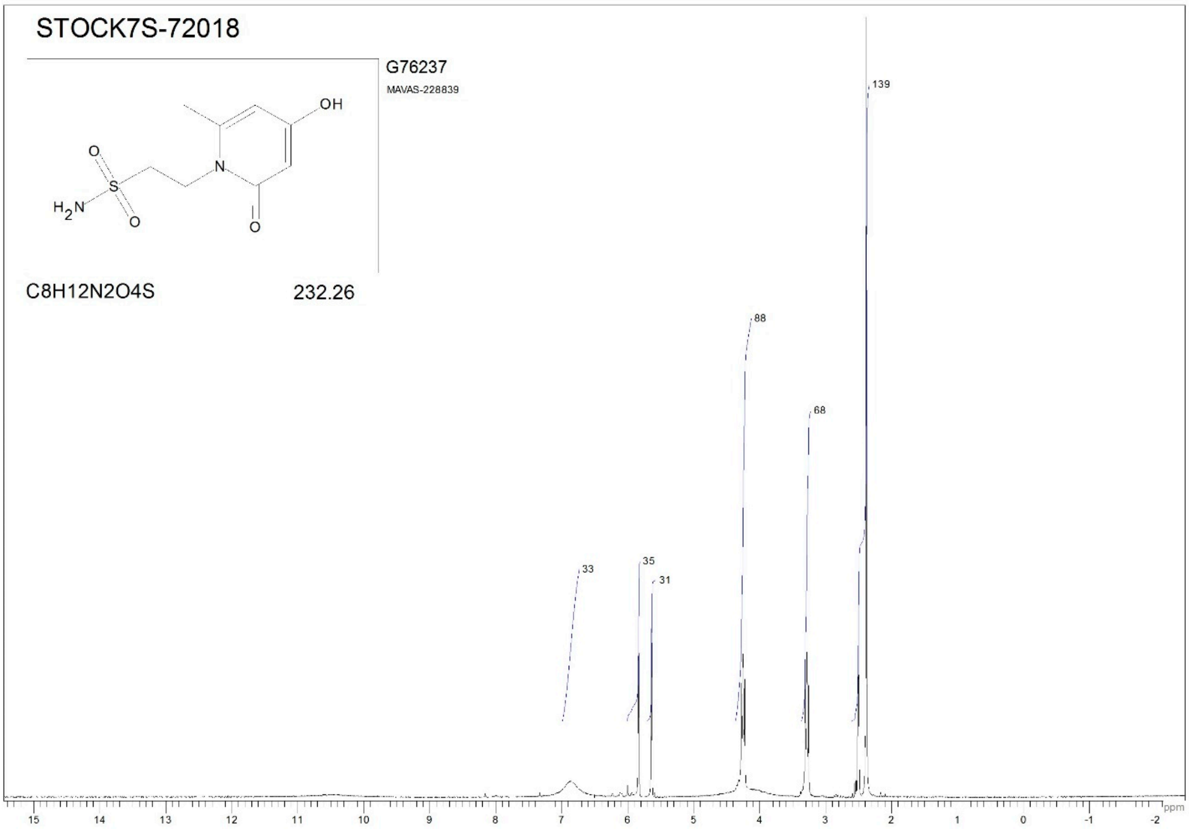
STOCK7S-72018
G76237
MAVAS-228839
OH
O
S
N
H_2N
O
O
C8H12N2O4S
232.26
139
88
68
33
35
31
15
14
13
12
11
10
9
8
7
6
5
4
3
2
1
0
-1
-2
ppm

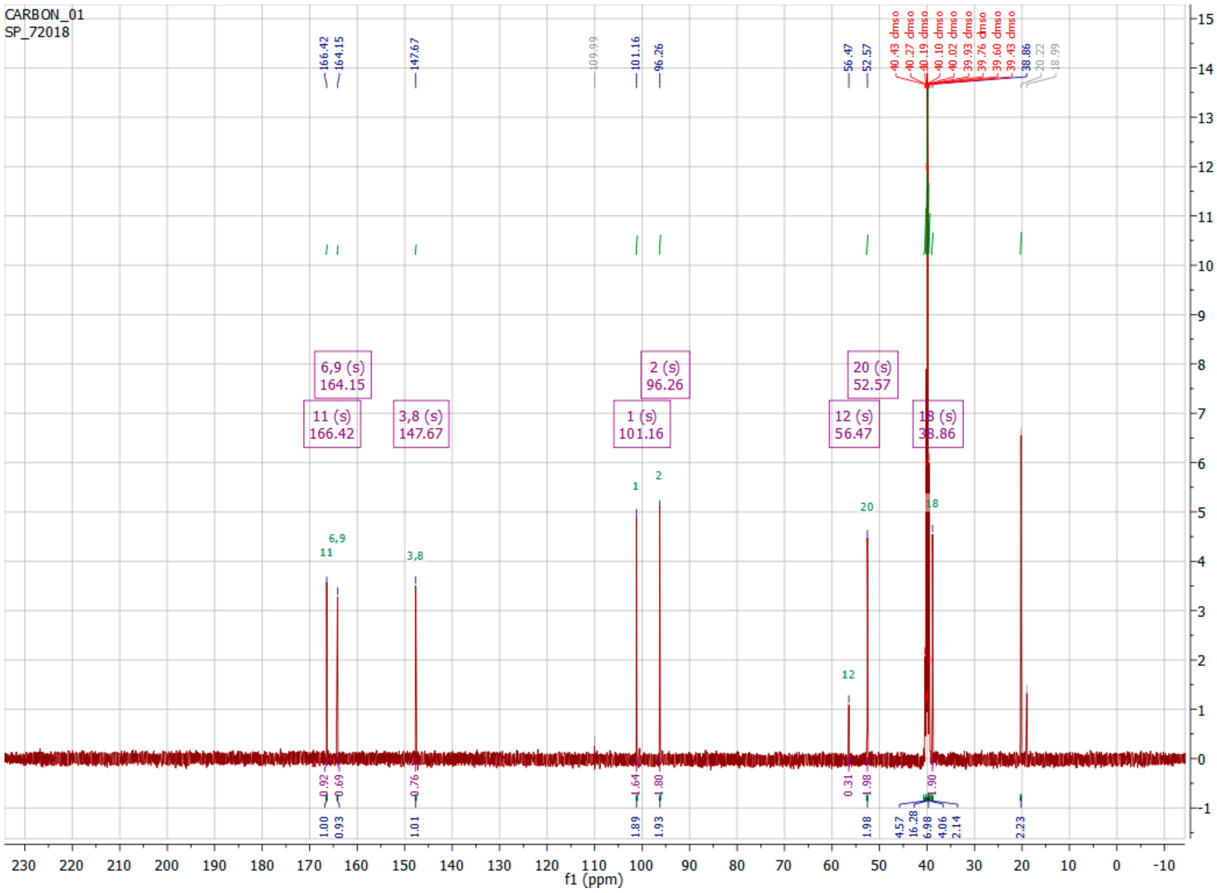
CARBON_01
SP_72018
6,9 (s)
164.15
11 (s)
166.42
3,8 (s)
147.67
2 (s)
96.26
1 (s)
101.16
20 (s)
52.57
12 (s)
56.47
18 (s)
38.86
f1 (ppm)

Compound 1j

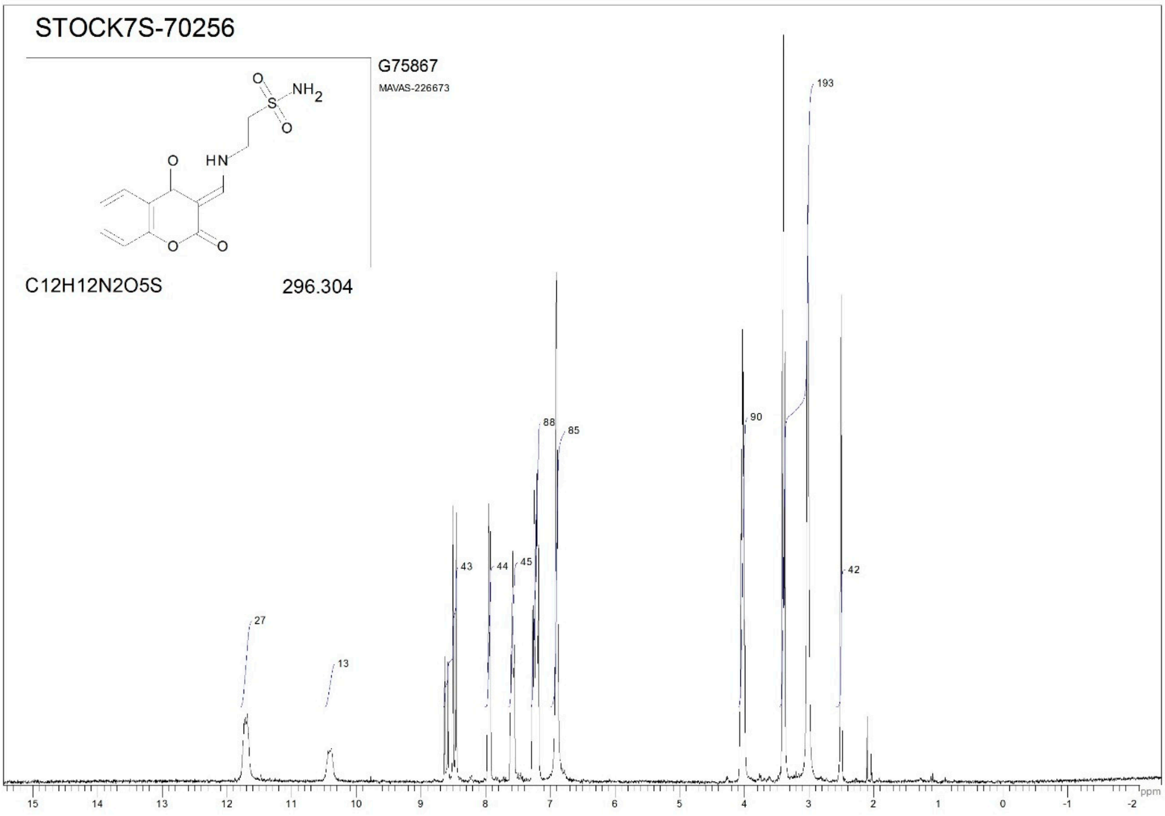
STOCK7S-70256
G75867
MAVAS-226673
C12H12N2O5S
296.304
ppm

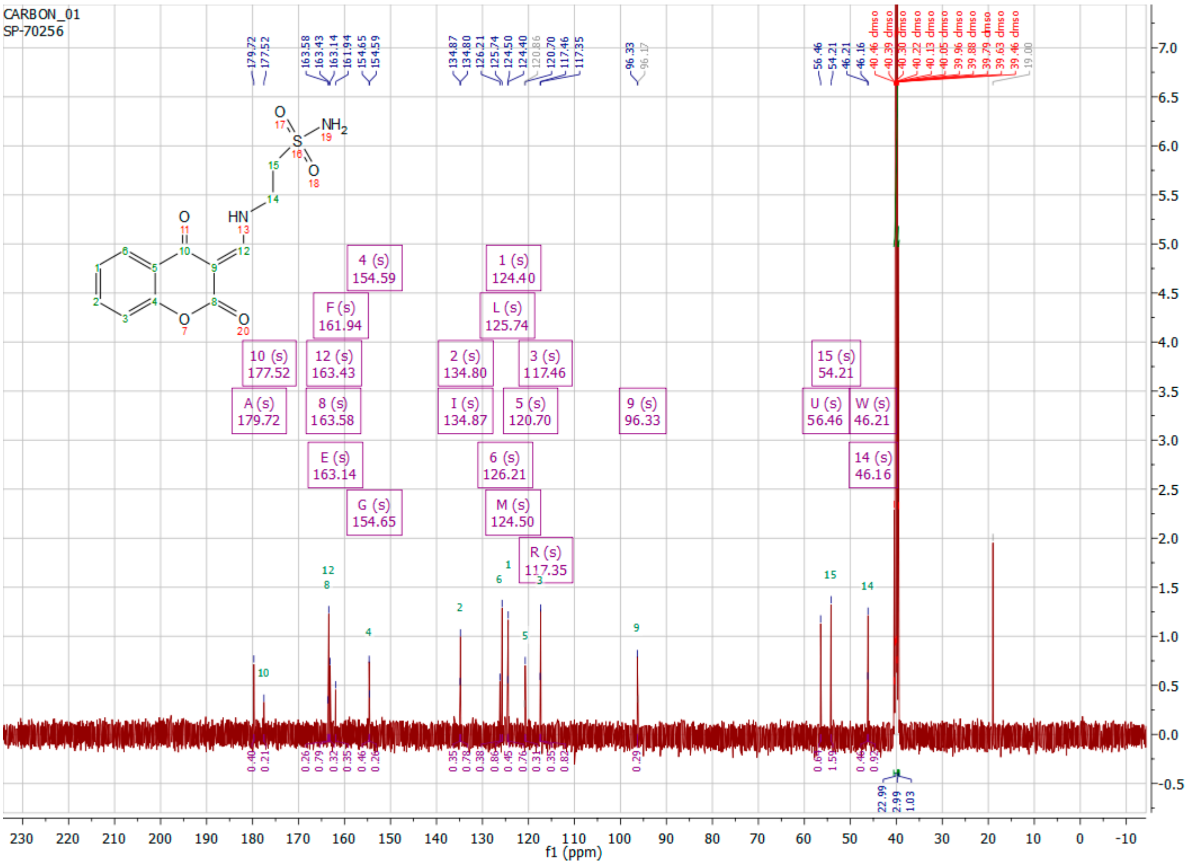
CARBON_01
SP-70256
f1 (ppm)

Compound 1k

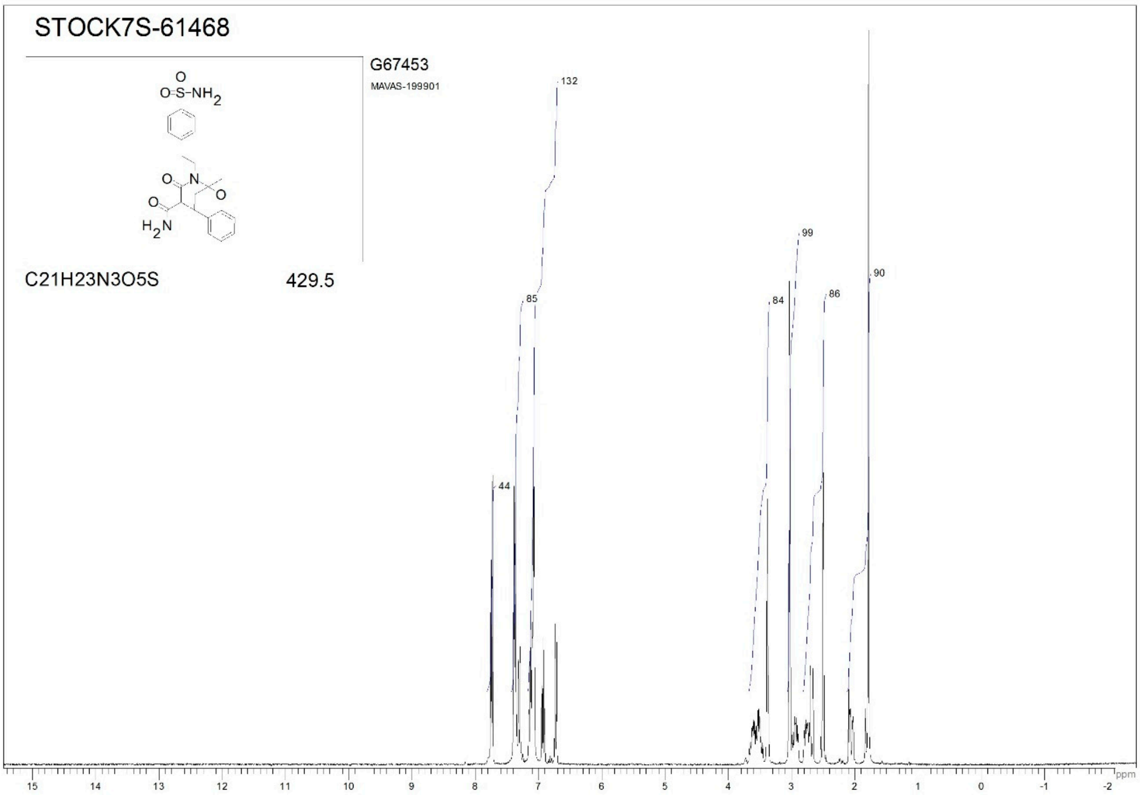
STOCK7S-61468
G67453
MAVAS-199901
C21H23N3O5S
429.5
ppm

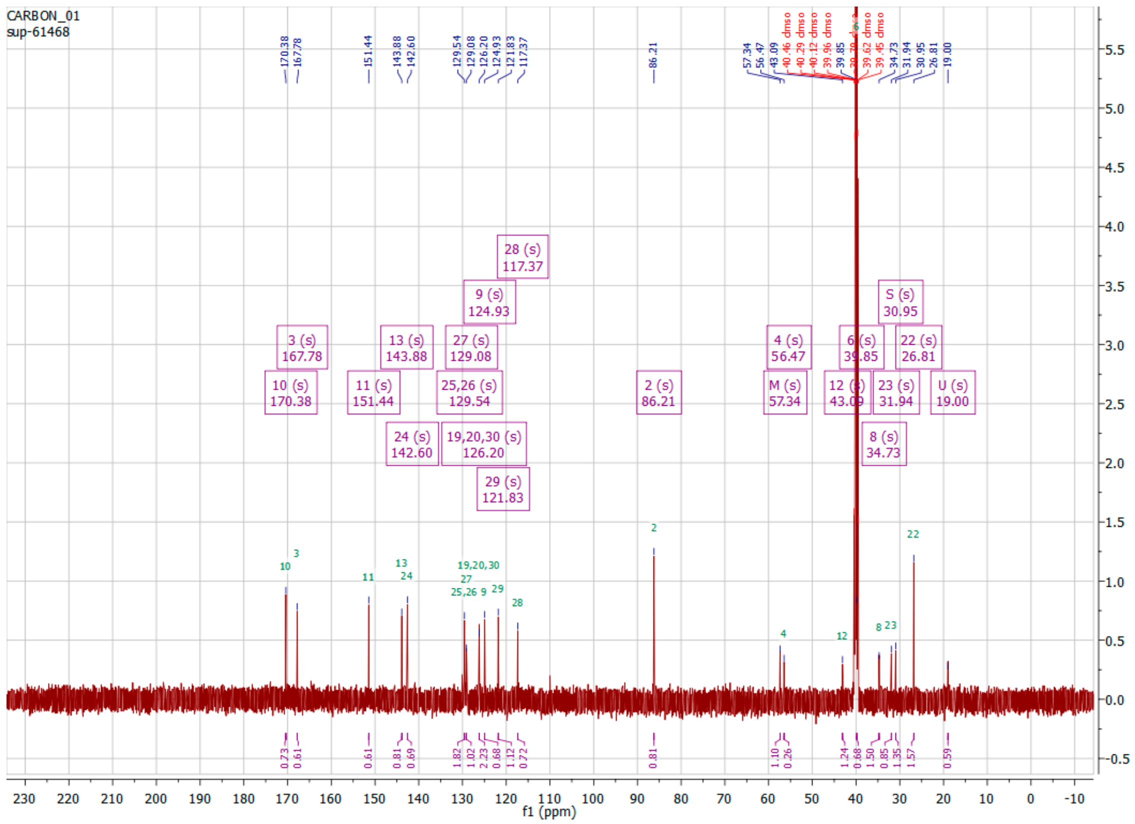

CARBON_01
sup-61468
3 (s) 167.78
10 (s) 170.38
11 (s) 151.44
13 (s) 143.88
24 (s) 142.60
27 (s) 129.08
25,26 (s) 129.54
19,20,30 (s) 126.20
9 (s) 124.93
28 (s) 117.37
29 (s) 121.83
2 (s) 86.21
4 (s) 56.47
M (s) 57.34
12 (s) 43.09
6 (s) 39.85
S (s) 30.95
22 (s) 26.81
23 (s) 31.94
8 (s) 34.73
U (s) 19.00
f1 (ppm)
